# Prokineticin 1 is a novel factor regulating porcine corpus luteum function

**DOI:** 10.1038/s41598-023-32132-3

**Published:** 2023-03-29

**Authors:** Monika Baryla, Ewelina Goryszewska-Szczurek, Piotr Kaczynski, Gianfranco Balboni, Agnieszka Waclawik

**Affiliations:** 1 Institute of Animal Reproduction and Food Research, Polish Academy of Sciences, Tuwima 10, 10-748 Olsztyn, Poland; 2 Department of Life and Environmental Sciences, University of Cagliari, Cittadella Universitaria, 09042 Monserrato, Cagliari Italy

**Keywords:** Reproductive biology, Animal physiology

## Abstract

Prokineticin 1 (PROK1) is a pleiotropic factor secreted by endocrine glands; however, its role has not been studied in the corpus luteum (CL) during pregnancy in any species. The present study aimed to investigate the contribution of PROK1 in regulating processes related to porcine CL function and regression: steroidogenesis, luteal cell apoptosis and viability, and angiogenesis. The luteal expression of *PROK1* was greater on Days 12 and 14 of pregnancy compared to Day 9. PROK1 protein expression during pregnancy increased gradually and peaked on Day 14, when it was also significantly higher than that on Day 14 of the estrous cycle. Prokineticin receptor 1 (*PROKR1*) mRNA abundance increased on Days 12 and 14 of pregnancy, whereas *PROKR2* elevated on Day 14 of the estrous cycle. PROK1, acting via PROKR1, stimulated the expression of genes involved in progesterone synthesis, as well as progesterone secretion by luteal tissue. PROK1–PROKR1 signaling reduced apoptosis and increased the viability of luteal cells. PROK1 acting through PROKR1 stimulated angiogenesis by increasing capillary-like structure formation by luteal endothelial cells and elevating angiogenin gene expression and VEGFA secretion by luteal tissue. Our results indicate that PROK1 regulates processes vital for maintaining luteal function during early pregnancy and the mid-luteal phase.

## Introduction

The corpus luteum (CL) is an important endocrine gland for pregnancy establishment in mammals^1^, and its main function is production of progesterone (P4), a hormone necessary for the development and maintenance of pregnancy. Progesterone synthesis is controlled by steroidogenic acute regulatory protein (STAR); side-chain cleavage cytochrome P450 (CYP11A1); and hydroxy-delta-5-steroid dehydrogenase, 3 beta- and steroid delta-isomerase 1 (HSD3B1).

The proper function of the CL is very important in pigs because it is a major source of progesterone during the entire pregnancy^2^. The lack of fertilization results in CL regression on days 14–16 post ovulation. During pregnancy, CL function is maintained by luteoprotective and luteotrophic factors such as estradiol-17β and prostaglandin E2^3^. Previous reports have indicated a high rate of embryo mortality in pigs during early pregnancy; however, the etiology of this phenomenon is not yet fully understood^4^. CL dysfunction has been suggested as one of the reasons for increased embryonic mortality in pigs^5^.

Several processes are involved in CL formation, luteal lifespan, and CL regression, including tissue remodeling, apoptosis, and angiogenesis associated with endothelial cells^6,7^. Angiogenesis in luteal tissue is tightly regulated during different phases of the CL lifespan. The main angiogenic factor in the CL is vascular endothelial growth factor A (VEGFA)^8^. However, prokineticin 1 (PROK1), known as endocrine gland-derived vascular endothelial growth factor (EG-VEGF), may also stimulate angiogenesis^9–11^. It is worth highlighting that PROK1 does not share sequence similarity with VEGFA^12^. Prokineticin 1 in mammals exerts pleiotropic, tissue-dependent effects, but the most recognized are the mitogenic effects on the endothelial cells of microvessels proximal to endocrine glands and on smooth muscle contractivity in intestines^9,13,14^. This short-amino acid chain protein belongs to the AVIT family and acts by binding to one of two receptors: prokineticin receptor 1 (PROKR1) and 2 (PROKR2)^15^. However, PROK1 has a higher affinity for PROKR1 than for PROKR2^16^. Both receptors act through the recruitment of different G-proteins (Gi, Gq and/or Gs) and can induce the signaling pathway of protein kinase C, mitogen-activated protein kinase, phosphoinositide 3 kinase or protein kinase B^17^. Nonetheless, PROK1 acting through one of its receptors can have different effects; for example, PROK1-PROKR1 signaling affects the growth of fetal endothelial cells, while PROK1-PROKR2 signaling induces endothelial cell permeability^18^.

The role of PROK1 in female reproductive function has been studied^19–23^. The expression of *PROK1* mRNA in the human endometrium increases during the peri-implantation period^24^. Our recent studies indicate that PROK1 and PROKR1 gene and protein expression in the endometrium and *PROK1* gene expression in the trophoblast are upregulated during implantation and early placentation in pigs^22,25^. PROK1, acting through PROKR1, is an embryonic signal mediator in the endometrium that participates in early pregnancy establishment by promoting angiogenesis, trophoblast cell proliferation, and adhesion and regulating the expression of genes involved in embryo–maternal interactions^22,25^. Moreover, PROK1 and its receptors (PROKRs) are expressed in ovarian follicles and corpora lutea in humans, cows, and rats^19,20,26–28^. Although PROK1 expression in the CL during the estrous cycle is dynamic^19,27,29^, the main role of PROK1 in the CL is still not fully understood. PROK1 effector cells in the corpus luteum are luteal endothelial cells and luteinized cells^20^. Based on the aforementioned studies, it may be supposed that PROK1 stimulates angiogenesis in the CL. However, the expression of PROK1 is strongest in the bovine and human CL during the late luteal stage or corpus luteum regression when angiogenesis declines^27,29^. Until now, only the expression profiles of the PROK1 gene, not the protein, have been evaluated in the corpus luteum during the estrous/menstrual cycles in cattle and humans. Moreover, there is a lack of data concerning the expression and role of PROK1 in the CL during early pregnancy in any species. PROK1 expression profiles and function in the porcine CL have not been studied until now.

We hypothesized that PROK1 regulates processes involved in controlling the luteal lifespan in pregnancy and the estrous cycle in pigs. Our objectives for the present study were (1) to evaluate PROK1 and its receptors’ (PROKR1 and PROKR2) gene and protein expression profiles in porcine corpora lutea collected during the luteal phase of the estrous cycle and corresponding days of pregnancy; (2) to determine the localization of PROK1 and its receptors’ protein expression in the porcine corpus luteum; and (3) to study the effects of PROK1 in the corpus luteum on major functions such as progesterone synthesis, luteal cell apoptosis and viability, and angiogenesis.

## Results

### The expression of PROK1 in the porcine corpus luteum is upregulated on Day 14 of pregnancy (Experiment 1)

**Figure 1:**
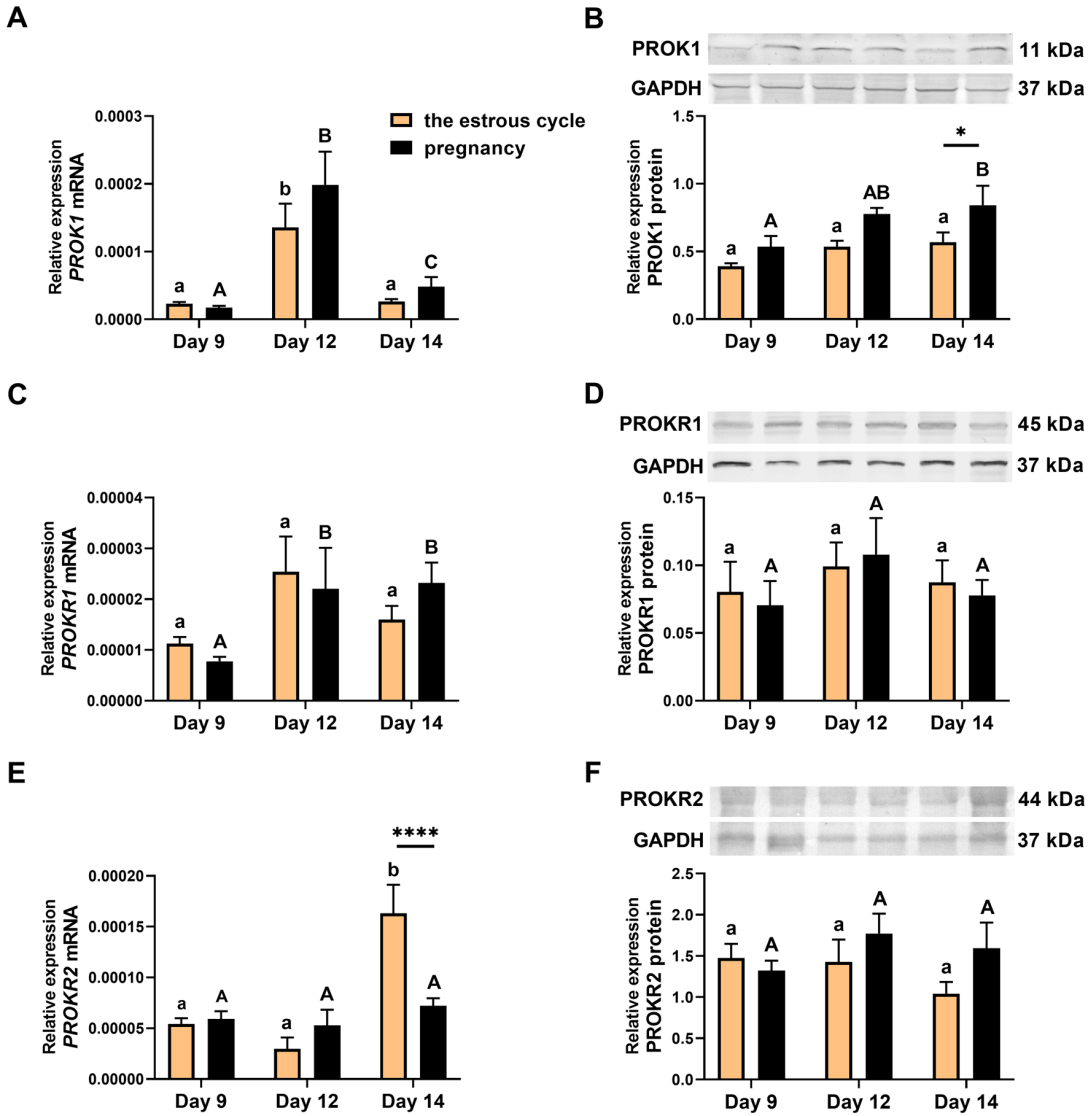
The expression profiles of prokineticin 1 (PROK1; **A** and **B**), prokineticin receptor 1 (PROKR1; **C** and **D**) and 2 (PROKR2; **E** and **F**) mRNA (**A**, **C**, and **E**) and protein (**B**, **D**, and **F**) in the porcine corpora lutea on Days 9, 12, and 14 of pregnancy and the estrous cycle. Representative samples of Western blots are shown in the upper panels (**B**, **D**, **F**). Uncropped images of full length Western blot membranes are shown in Supplementary Fig. 2. Data are presented as means ± SEM. Bars with different letters differ significantly (*p* < 0.05) within the estrous cycle (lowercase letters) and pregnancy (capital letters). Asterisks indicate statistical differences between reproductive statuses (pregnant vs. cyclic) on corresponding day of the estrous cycle and pregnancy (*p < 0.05; ***p < 0.001).

The expression profiles of PROK1 and its receptors in porcine corpora lutea were compared during the mid-luteal phase with the highest luteal activity (Days 9 and 12 of the estrous cycle), the beginning of luteolysis on Day 14 of the estrous cycle and the corresponding days of pregnancy (Day 9 and when CL function is rescued from regression on Days 12 and 14 of pregnancy; Fig. 1). We found a significant effect of the estrous cycle/pregnancy day on the expression of the PROK1 gene and protein and on the *PROKR1* and *PROKR2* genes (*p* < 0.01; Fig. 1A–E). A reproductive status effect (cyclic/pregnant) was found on PROK1 protein expression (*p* < 0.0001; Fig. 1B). An interaction between the effect of reproductive status and the effect of the estrous cycle/pregnancy day was detected for the gene expression of *PROKR2* (*p* < 0.01; Fig. 1E).

*PROK1* mRNA expression was higher on Day 12 of the estrous cycle and pregnancy than on Days 9 and 14 of the estrous cycle (*p* < 0.001). *PROK1* gene expression was greater on Days 12 and 14 of pregnancy than on Day 9 of pregnancy (*p* < 0.0001 and *p* < 0.05, respectively; Fig. 1A). PROK1 protein expression during pregnancy increased gradually and reached the highest level on Day 14 (vs. Day 9; *p* < 0.05, and vs. Day 14; *p* < 0.0001, Fig. 1B). Furthermore, PROK1 protein expression was significantly higher on Day 14 of pregnancy than on Day 14 of the estrous cycle (*p* < 0.05; Fig. 1B). The expression of the *PROKR1* gene was higher on Days 12 and 14 of pregnancy than on Day 9 of pregnancy (*p* < 0.05; Fig. 1C), whereas *PROKR2* gene expression was upregulated on Day 14 of the estrous cycle compared with Days 9 and 12 of the estrous cycle (*p* < 0.001 and *p* < 0.0001, respectively) and Day 14 of pregnancy (*p* < 0.01; Fig. 1E). No significant differences in PROKR1 and PROKR2 protein expression were found between Days 9 and 14 of the estrous cycle or pregnancy (Fig. 1D and F). The concentration of P4 in plasma from jugular vein blood was elevated on Day 14 of pregnancy compared to Day 14 of the estrous cycle (Supplementary Fig. 1).

### The PROK1, PROKR1, and PROKR2 proteins are expressed in steroidogenic cells and blood vessels of the porcine corpus luteum (Experiment 2)

**Figure 2:**
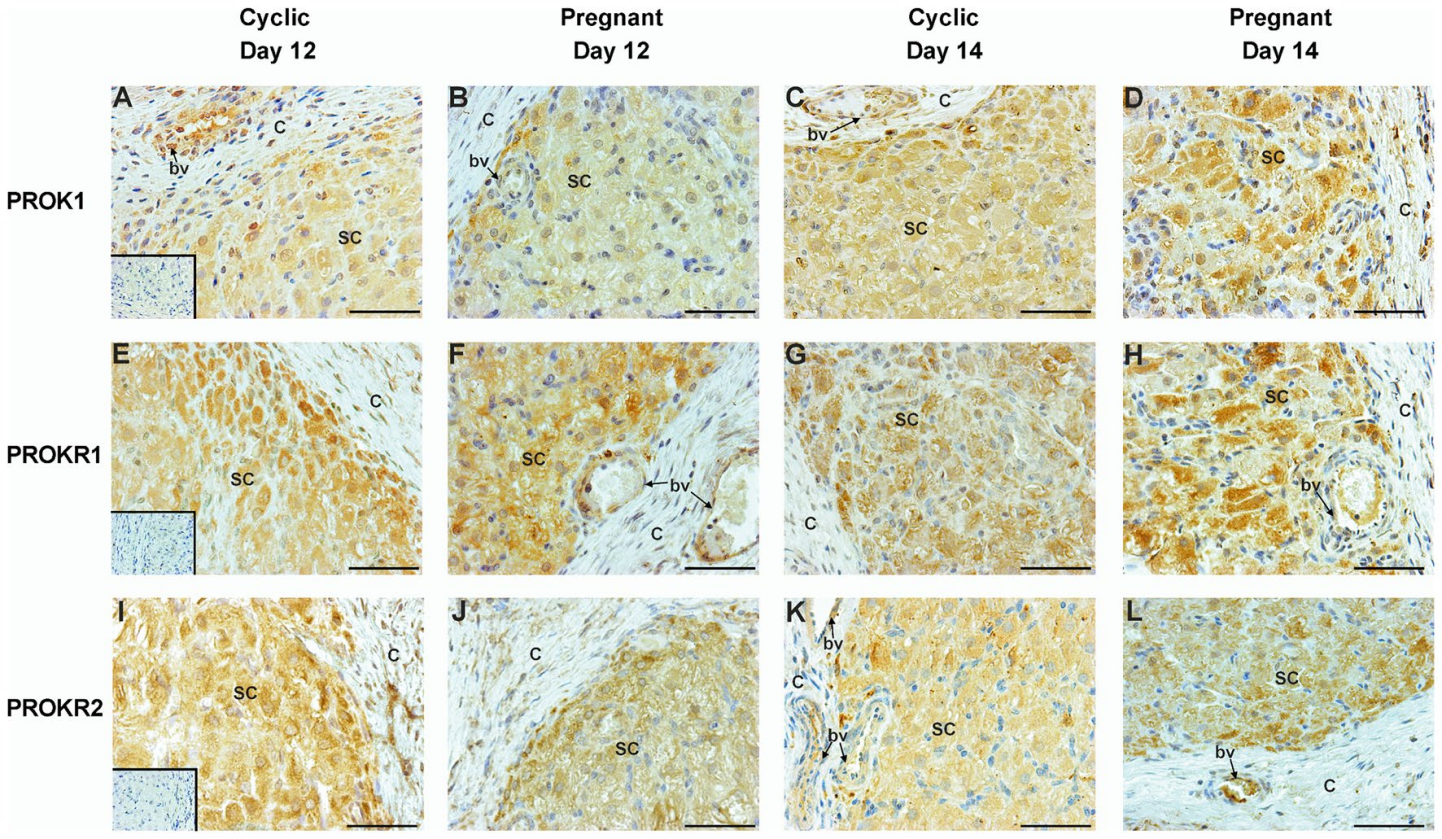
Immunolocalization of prokineticin 1 (PROK1; **A**–**D**), prokineticin receptor 1 (PROKR1; **E**–**H**), and 2 (PROKR2; **I**–**L**) in the porcine corpora lutea on Day 12 (**A**, **B**, **E**, **F**, **I**, and **J**) and 14 (**C**, **D**, **G**, **H**, **K**, and **L**) of the estrous cycle (**A**, **C**, **E**, **G**, **I**, and **K**) and pregnancy (**B**, **D**, **F**, **H**, **J**, and **L**). Positive signals of all studied proteins were detected in luteal steroidogenic cells (SC), and endothelial cells that forms blood vessels (“bv” with arrow). A week signal or no signal is shown in theca derived luteal capsule (**C**). All sections were counterstained with hematoxylin. The negative control is presented in the down-left corners of A, I (Normal rabbit IgG), and E panels (antibodies with the blocking peptide). Scale bar represents 50 µm.

The protein expression of PROK1 (Fig. 2A–D), PROKR1 (Fig. 2E–H), and PROKR2 (Fig. 2I–L) in the CL during the estrous cycle and pregnancy (Days 12 and 14) was detected mainly in steroidogenic cells (SC) and luteal blood vessels (bv). No staining or weak staining for PROK1, PROKR1, and PROKR2 was observed in the theca-derived luteal capsule (C) of the porcine CL (Fig. 2). The specificity of the antibodies used in immunohistochemical analyses was confirmed using negative controls (PROK1 and PROKR2—normal rabbit IgG; PROKR1—blocking peptide). Microphotographs of the negative controls are presented in the lower-left parts of Panels A, E, and I in Fig. 2.

### PROK1 increases the expression of genes involved in progesterone synthesis and enhances progesterone production by porcine luteal explants in vitro* (Experiment 3)*

**Figure 3:**
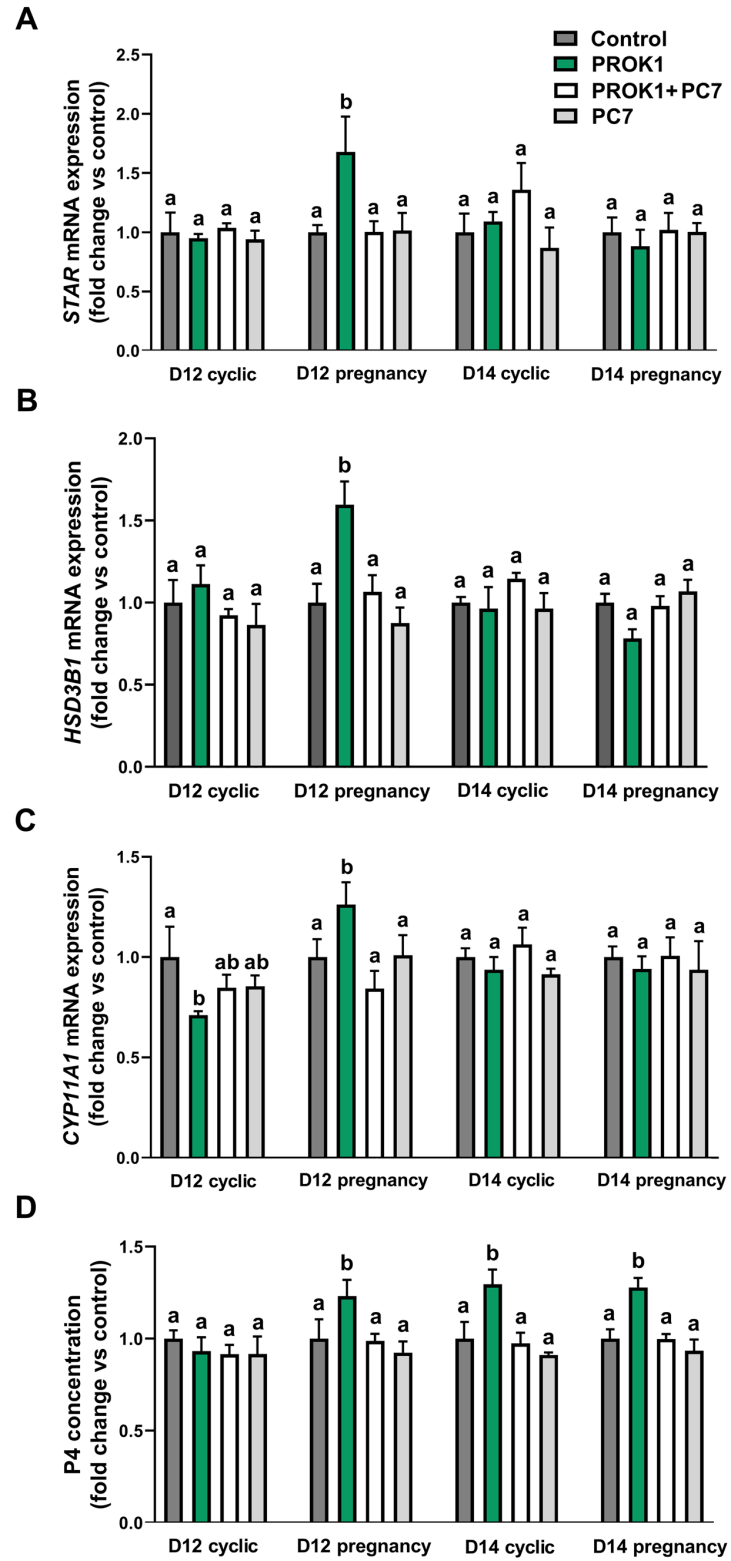
The effect of prokineticin 1 (PROK1) on expression of genes involved in progesterone (P4) synthesis and on P4 production by the porcine luteal explants in vitro on Day 12 and 14 of estrous cycle or pregnancy: (**A**) steroidogenic acute regulatory protein (*STAR*), (**B**) hydroxy-delta-5-steroid dehydrogenase, 3 beta- and steroid delta-isomerase 1 (*HSD3B1*), (**C**) cytochrome P450 family 11 subfamily A member 1 (*CYP11A1*) genes and (**D**) P4 secretion. Precision-cut luteal slices were incubated with the control (vehicle) or PROK1 (40 nM) for 18 h in the presence/absence of prokineticin receptor 1 (PROKR1) antagonist (PC7; 10 µM). Data are presented as means ± SEM. Different lowercase letters (a, b) indicate statistical significant differences between treatments within groups on Days 12 (D12) and 14 (D14) of the estrous cycle or pregnancy (*p* < 0.05).

The effects of PROK1 on the luteal expression of genes responsible for progesterone synthesis and on the P4 synthesis by CL explants were studied using a precision-cut luteal slice in vitro model. An effect of day of the estrous cycle/pregnancy and an interaction between the effects of treatment and the effect of day of the estrous cycle/pregnancy were detected for the gene expression of *STAR* (*p* < 0.05; Fig. 3A), *HSD3B1* (*p* < 0.01; Fig. 3B), and *CYP11A1* (*p* < 0.01; Fig. 3C) and for secretion of progesterone (*p* < 0.05; Fig. 3D). Moreover, an effect of treatment on progesterone secretion was found (*p* < 0.0001; Fig. 3D).

Prokineticin 1 elevated the expression of the *STAR* and *HSD3B1* genes in luteal explants collected on Day 12 of pregnancy (*p* < 0.0001; Fig. 3A, and B). Interestingly, we found stimulating effects of PROK1 on luteal *CYP11A1* expression in CL explants collected on Day 12 of pregnancy (*p* < 0.05; Fig. 3C) compared to the control, whereas in CL explants collected on Day 12 of the estrous cycle, PROK1 reduced the expression of *CYP11A1* (*p* < 0.05; Fig. 3C). The results of the radioimmunoassay indicated that PROK1 elevated the concentration of progesterone in medium from luteal explants collected on Day 12 of pregnancy (*p* < 0.05), Day 14 of the estrous cycle (*p* < 0.05), and Day 14 of pregnancy (*p* < 0.01; Fig. 3D). The stimulating effects of PROK1 were abolished by a PROKR1 antagonist (PC7). The P4 concentration (mean ± SEM) in the control medium was 1717 ng/mL ± 121 on Day 12 of the estrous cycle, 1347 ng/mL ± 182 on Day 12 of pregnancy, 1314 ng/mL ± 219 on Day 14 of the estrous cycle, and 1621 ng/mL ± 213 on Day 14 of pregnancy.

**Figure 4:**
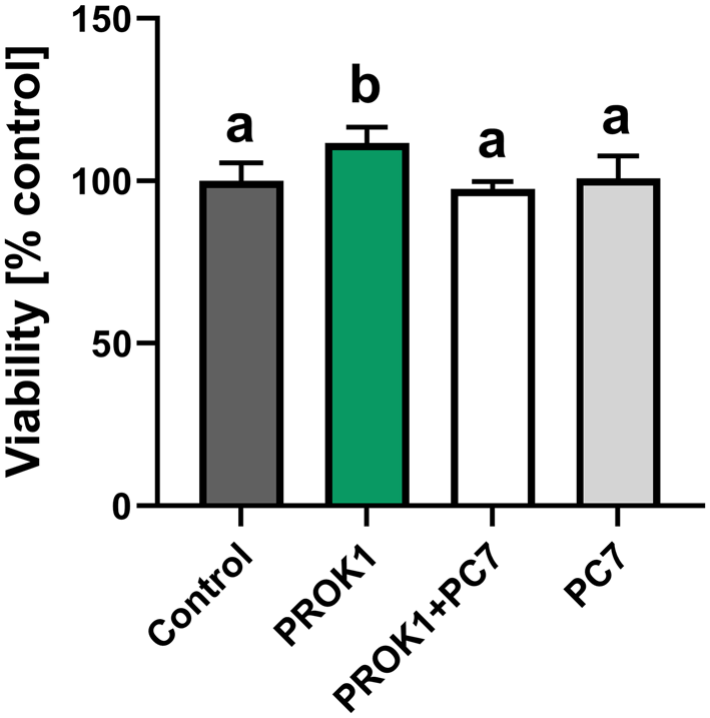
The viability of luteal explants after in vitro treatment analyzed by Alamar Blue assay. Precision-cut luteal slices were incubated with control (vehicle) or PROK1 (40 mM) for 18 h in the presence/absence of prokineticin receptor 1 (PROKR1) antagonist (PC7; 10 µM). Data are presented as means of the fold change versus control (100%) ± SEM. Different lowercase letters (a, b) indicate statistical significant differences between treatments.

To determine the effects of the incubation time and treatment on the viability of the luteal slices, an Alamar Blue assay was performed. The metabolic activity of CL explants was not significantly different between 0 and 18 h of incubation (100 ± 7.29% vs. 91.32 ± 0.87%). In addition, none of the treatments used decreased the tissue explant viability after 18 h of incubation compared to the control (Fig. 4). The antagonist for PROKR1 alone or in combination with PROK1 did not have any effect on tissue explant viability. However, PROK1 slightly increased the viability of CL explants compared to the control (*p* < 0.05; Fig. 4).

### PROK1 reduces luteal cell apoptosis and increases the viability of luteal cells (Experiment 4)

Isolated primary luteal cells were characterized (Supplementary Fig. 3). We confirmed the presence of the luteal cell marker HSD3B1 (Supplementary Fig. 3A, and C). The specificity of the antibodies used in immunofluorescence analyses was confirmed using the negative control (Supplementary Fig. 3D).

**Figure 5:**
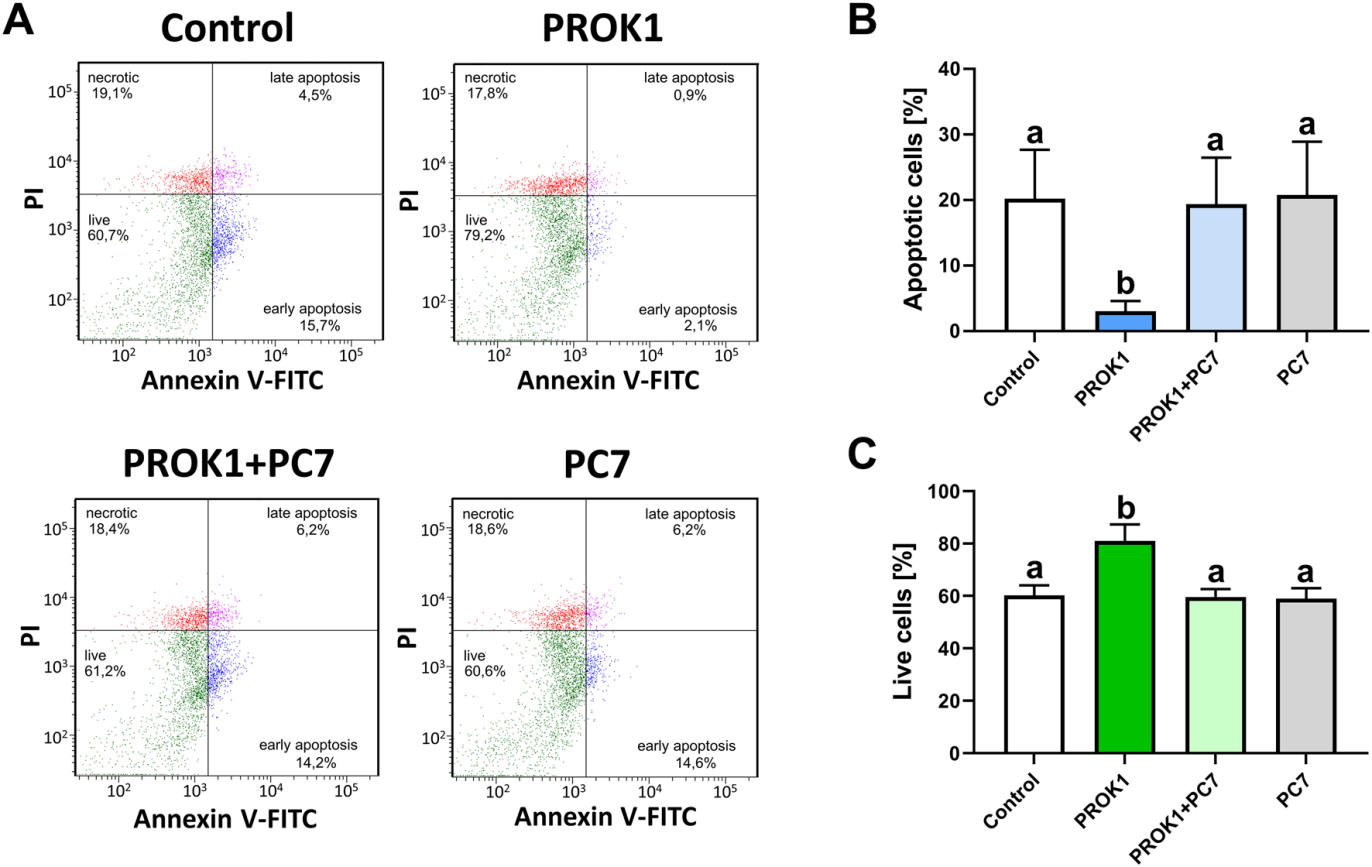
The effect of prokineticin 1 (PROK1) on apoptosis and viability of the porcine luteal cells collected on Day 12 of the estrous cycle. Three cell phenotypes were distinguished: live (annexin V-negative/PI-negative), apoptotic (sum of early apoptotic—annexin V-positive/PI-negative and late apoptotic—annexin V-positive/PI-positive), and necrotic cells (annexin V-negative/PI-positive). (**A**) Representative scatter plots of PI (y-axis) vs. annexin V (x-axis) with percentages of individual cell phenotypes are shown in panels on the right of the graphs; (**B**) apoptotic cells, and (**C**) live cells. Isolated cells were treated with the control (vehicle) or PROK1 (40 nM) in the presence or absence of prokineticin receptor 1 (PROKR1) antagonist (PC7; 1 µM). The assay was performed by using fluorescence-activated single-cell sorting based on phenotypes detected by flow cytometry. Data are presented as means ± SEM. Different lowercase letters (a, b) indicate statistically significant differences between groups (*p* < 0.05).

FITC-Annexin V and propidium iodide staining were used to determine the effect of PROK1 on porcine luteal cell apoptosis and viability. PROK1 significantly reduced the percentage of apoptosis compared with the control (*p* < 0.05, Fig. 5B). Moreover, PROK1 significantly increased cell viability compared with the control (*p* < 0.05, Fig. 5C). These stimulating effects of PROK1 on apoptosis and the viability of luteal cells were diminished using a PROKR1 antagonist (PC7). No effect of PROK1 treatment on the number of necrotic cells was detected (Fig. 5A).

### PROK1 stimulates angiogenesis in the porcine corpus luteum in vitro* (Experiment 5)*

#### PROK1 promotes capillary-like network formation by luteal endothelial cells

We characterized isolated primary luteal endothelial cells (Supplementary Fig. 4) and confirmed the presence of the endothelial cell marker von Willebrand factor (vWF) in the isolated cells (Supplementary Fig. 4A, D, E, and H). The specificity of the antibodies used in immunofluorescence analyses was confirmed using a negative control—normal rabbit IgG (Supplementary Fig. 4I).

**Figure 6:**
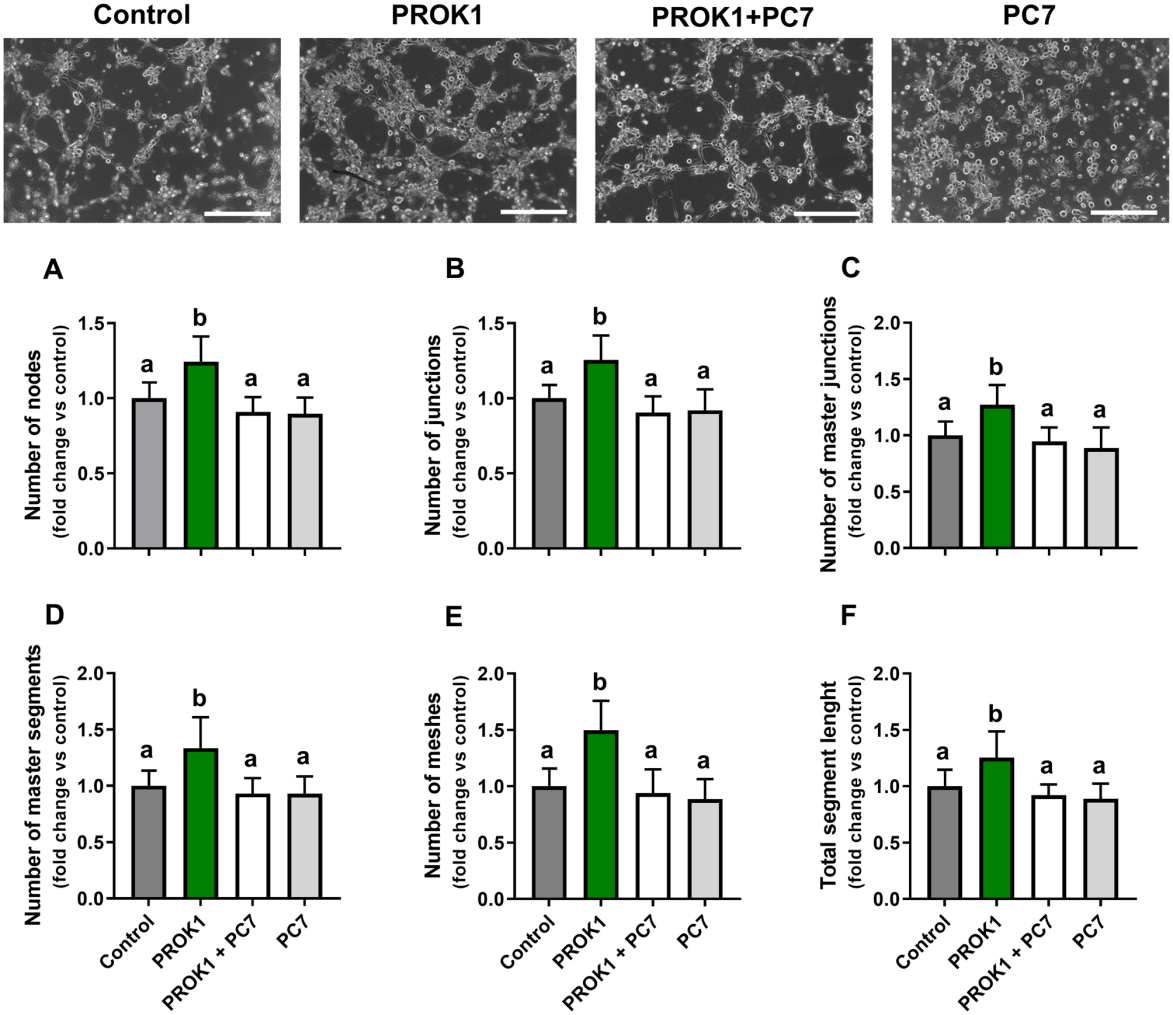
The prokineticin 1 (PROK1) induces capillary-like structure formation by porcine primary luteal endothelial cells isolated on Day 12 of the estrous cycle. The effects of PROK1 (40 nM) on selected parameters describing angiogenesis, such as: (**A**) the number of nodes, (**B**) number of junctions, (**C**) number of master junctions, (**D**) number of master segments, (**E**) number of meshes, and (**F**) total segment length were evaluated in the presence or absence of the prokineticin receptor 1 (PROKR1) antagonist (PC7, 1 µM). Representative photographs of the capillary-like structure formation assay for porcine primary luteal endothelial cells are shown in panels above graphs. Data are presented as means ± SEM of the fold change versus control, different lowercase letters (a, b) indicate statistically significant differences (*p* < 0.05). The scale bar at the lower-right part of all photographs represents 25 µm.

The capillary-like structure formation assay for porcine primary luteal endothelial cells isolated from the corpus luteum collected from gilts on Day 12 of the estrous cycle revealed that PROK1 increased the numbers of nodes, junctions, master junctions, master segments, meshes, pieces, segments, and isolated segments as well as the total master segment length, total mesh area, total length, total branching, segment length, branching interval, mesh index, and mean mesh size (*p* < 0.05; Fig. 6A–F, and Supplementary Fig. 5B–E, G–I, and L–N). The significant effects of PROK1 on the analyzed parameters describing angiogenesis were abolished by a PROKR1 antagonist (PC7).

#### PROK1 upregulates ANG gene expression and VEGFA secretion in luteal tissue

**Figure 7:**
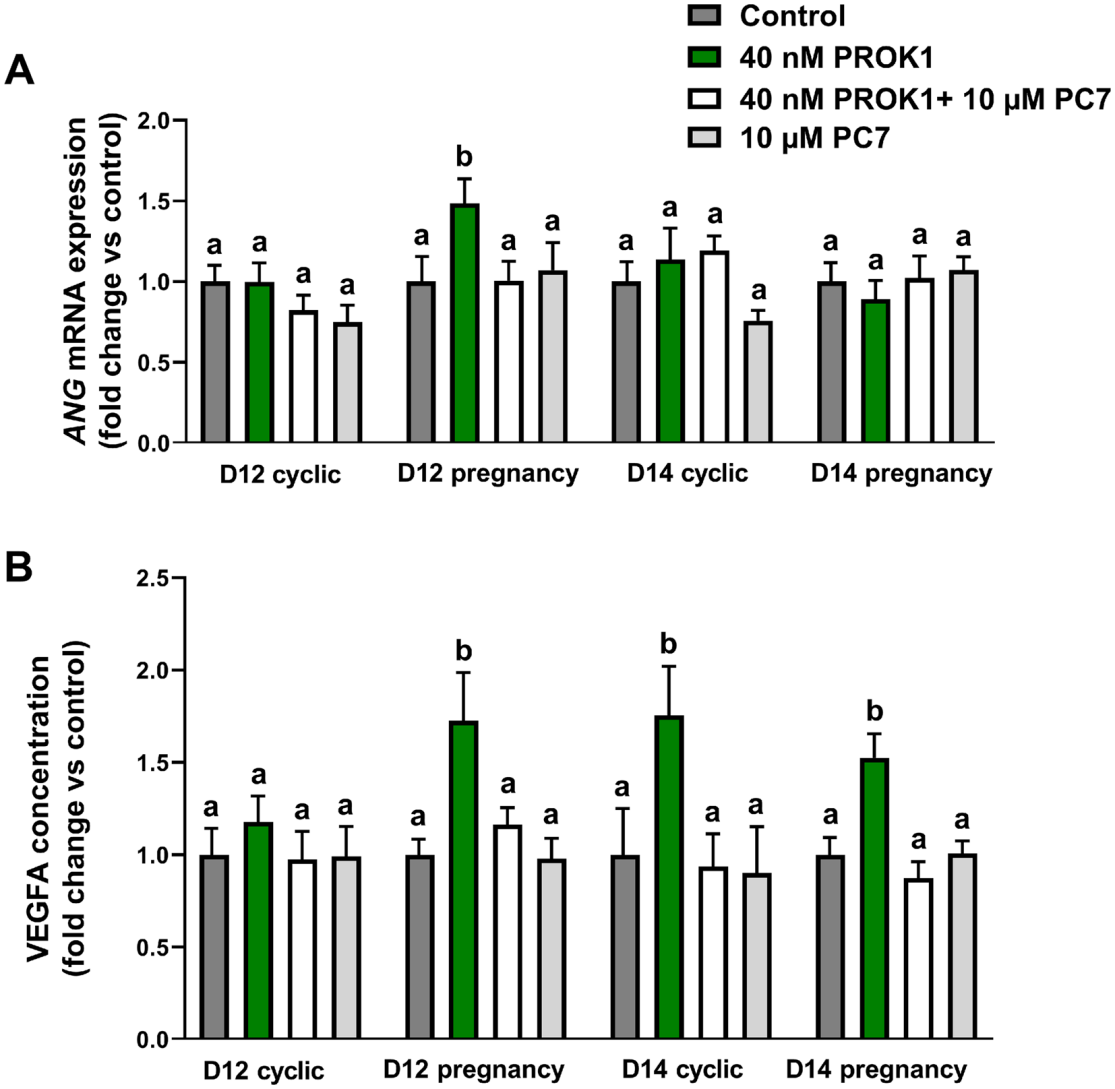
The effect of prokineticin 1 (PROK1) on (**A**) mRNA expression of angiogenin (*ANG*) and (**B**) on vascular endothelial growth factor A (VEGFA) secretion. Precision-cut luteal slices were incubated with control (vehicle) or PROK1 (40 mM) for 18 h in the presence/absence of prokineticin receptor 1 (PROKR1) antagonist (PC7; 10 µM). Data are presented as means ± SEM. Different lowercase letters (a, b) indicate statistical significant differences between treatments within groups on Days 12 (D12) and 14 (D14) of the estrous cycle or pregnancy.

We used precision-cut luteal slices in the in vitro model to examine whether PROK1 may affect the expression of genes associated with angiogenesis and VEGFA secretion. We found effects of the treatment only on *ANG* gene expression (*p* < 0.05; Fig. 7A) and VEGFA secretion (*p* < 0.0001; Fig. 7B). PROK1 increased the abundance of *ANG* mRNA in luteal explants collected on Day 12 of pregnancy (*p* < 0.01; Fig. 7A). These stimulating effects of PROK1 were abolished using PC7. No effects of PROK1 treatment were detected on the gene expression of *ANGPT2*, *FGF2*, *FLT1* and *KDR* in luteal explants (Supplementary Fig. 6A–D).

PROK1 elevated the secretion of VEGFA into the culture medium of luteal explants collected on Day 12 of pregnancy (*p* < 0.05) and on Day 14 of the estrous cycle and pregnancy (*p* < 0.01 and *p* < 0.05, respectively; Fig. 7B). The stimulating effects of PROK1 were inhibited by PC7. The VEGFA concentration (mean ± SEM) in the control medium was 9.54 pg/mL ± 2.45 on Day 12 of the estrous cycle, 9.49 pg/mL ± 3.14 on Day 12 of pregnancy, 6.31 pg/mL ± 2.13 on Day 14 of the estrous cycle, and 5.93 pg/mL ± 2 on Day 14 of pregnancy.

## Discussion

Our study is the first report on the role of PROK1 and its receptors in the porcine corpus luteum during the mid-luteal (high luteal activity) and late-luteal (decreased luteal activity) phases of the estrous cycle and the corresponding days of pregnancy, including the period of luteal rescue from regression in response to embryonic estradiol (Days 12 and 14 of pregnancy). We combined our ex vivo approach with an in vitro model of ultrathin luteal tissue explants (180 µm), which is more physiological than using one particular population of cells isolated from the CL. We used pigs as models for studying the mechanisms underlying CL physiology because, among others, in the pig as a multiparous species, several CL are usually formed in each ovary, providing an excellent source of material for multiple analyses in tissue studies. Moreover, the cellular mechanisms involved in the regulation of reproductive processes can be common among species with different types of placentation despite of species-specific differences in the establishment of pregnancy^30–32^. Hence, the presented results may be applicable to the CL of other species.

Our results revealed that luteal expression of *PROK1* mRNA is elevated in the mid-luteal phase of the estrous cycle, which aligns with the findings of previous studies on the bovine and human corpora lutea^19,27,29^. However, in the late luteal phase, the gene expression of *PROK1* was decreased in the porcine CL, whereas in cattle, it is still elevated^27^. It has been suggested that the increased expression of the *PROK1* and *PROKR1* genes in the bovine CL at the late luteal phase may be related to the involvement of these genes in recruiting monocytes to the regressing CL^27^. Therefore, these interspecies discrepancies may be the difference in the stage of regression between these two species. The exact stage of regression is difficult to assess without administration of exogenous prostaglandin F2α and collection of timed luteal samples. However, we did not perform such an analysis because we decided to show the expression of PROK1 and its receptors under physiological conditions (the estrous cycle and early pregnancy). Nonetheless, our results are consistent with those of earlier studies showing that stress conditions such as cell starvation, hypoxia and high levels of tumor necrosis factor, which also occur during luteolysis, result in markedly elevated levels of *PROKR2* mRNA and do not affect the expression of PROK1 in bovine endothelial cells of CL origin^26^. Interestingly, PROK1 acting through PROKR2 may exert antiapoptotic effects on endothelial cells in the bovine CL^26^.

Until now, only gene expression for the PROK1–PROKR system has been reported for the CL in other species. In our study we demonstrated the protein expression of PROK1 and its receptors. The profiles of PROK1 mRNA and protein expression were relatively parallel, with somewhat of a shift in protein expression. Both the mRNA and protein levels were, however, greater on Day 14 of pregnancy than on Day 9 of pregnancy. The expression of *PROKR1* and *PROKR2* mRNA was regulated during the estrous cycle and pregnancy, but PROKR1 and PROKR2 protein expression did not change significantly in the studied periods. The discrepancies between the mRNA and protein abundances for PROK1 and its receptors in the porcine CL may have resulted from the fact that transcription and translation do not always have a linear relationship^33^. Protein synthesis from a certain copy number of mRNA molecules may be either enhanced or repressed depending on different mechanisms, as described elsewhere^34^. Moreover, discrepancies between mRNA and protein expression can be caused by the time shift between mRNA and protein synthesis. Most likely, the maximum *PROK1* mRNA expression on Day 12 resulted in a peak PROK1 protein expression slightly later on Day 14 of pregnancy. However, because of the luteolytic events occurring in the porcine CL shortly after Day 12 of the estrous cycle, the increase in *PROK1* mRNA could not be observed on Day 14 of the estrous cycle. Regarding PROKR2, it is also possible that the increase in *PROKR2* mRNA abundance observed on Day 14 of the estrous cycle could have resulted in elevated protein expression later than on Day 14 of the estrous cycle. Another explanation for this discrepancy may be the fact that Western blotting is less accurate than qPCR, and subtle changes may have been hidden.

Although there are numerous reports on PROK1 in the CL in other species, our study is the first concerning PROK1 and its receptor gene and/or protein expression profiles in the CL during early pregnancy in any species. The abundance of the PROK1 transcript and protein was elevated in the luteal tissue during pregnancy at the maternal recognition of pregnancy and implantation period. This finding suggests that the role of PROK1 in the corpus luteum may be especially significant during pregnancy in luteoprotective/antiluteolytic mechanisms, ensuring prolonged CL function. Our results correspond with reports of increased PROK1 expression in the pregnant endometrium at the time of embryo implantation in pigs^22^ and women^35,36^.

We demonstrated that PROK1 prevented porcine luteal cells from undergoing apoptosis; therefore, PROK1 supports the function of the corpus luteum during early pregnancy, when elevated levels of PROK1 are observed. Apoptosis, a type of programmed cell death^7^, has been primarily associated with luteal regression^37^. However, apoptosis is also an important process for developing CL, in which dynamic changes in tissue remodeling occur^7^. Moreover, the significant role of the apoptotic process in controlling the immune response (i.e., the deletion of immune cells recognizing self-antigens and cytotoxic killing) has been described^38^. Accordingly, the antiapoptotic effect of PROK1 has been demonstrated in different cell types—endothelial cells of CL origin in cattle^26^.

The expression of PROK1 and its receptors PROKR1 and PROKR2 was localized in porcine steroidogenic cells and blood vessels in the porcine CL during the mid- and late luteal phases and early pregnancy. This finding suggests that PROK1 acts in an autocrine and/or paracrine manner in porcine luteal tissue. Similar results for PROK1 localization have been described for bovine^27^ and human corpora lutea^19,29^. Colocalization of PROK1 and PROKRs in blood vessels and steroidogenic cells implies the significant effect of PROK1 on crucial processes related to corpus luteum function: steroidogenesis and angiogenesis.

We found that prokineticin 1 stimulated the secretion of P4 by CL explants collected on Day 12 of pregnancy and on Day 14 of pregnancy and the estrous cycle. On the other hand, the expression of genes involved in steroidogenesis (*HSD3B1*, *STAR*, and *CYP11A1*) was found to be upregulated by PROK1 only on Day 12 of pregnancy. This could have been caused by measurement of the gene expression and P4 secretion at the same time-point. The increase in P4 might have occurred later in the tissue collected on these specific days. Another explanation is that PROK1 regulates the synthesis of P4 in the CL on Day 14 of pregnancy/or the estrous cycle via mechanisms other than direct regulation, such as cholesterol or LDL uptake or storage, progesterone metabolism or action of PROK1-induced VEGFA. Another possible explanation for the increased P4 secretion by the luteal tissue collected from the regressing CL on Day 14 of the estrous cycle is that the potential effect of PROK1 was maintained when the tissue was separated from the luteolytic signals present in vivo. The lack of suppression of P4 secretion by luteal tissue collected on Day 14 of the estrous cycle may indicate a limitation of this in vitro model; hence, our conclusions on the role PROK1 in porcine CL during luteolysis should be interpreted with caution. However, the results on gene expression and P4 secretion were consistent for luteal tissue collected on Day 12 of pregnancy. Our results show the effects of PROK1 on the expression of genes involved in P4 synthesis and on P4 production by luteal tissue, which thus far have not been studied in any species. A previous study using the luteinized human granulosa cell line SVOG implied that steroidogenesis and the increased transcript abundance of the PROK1 gene could be positively correlated; however, no direct experiments have been performed to demonstrate this relationship^20^. Our findings are consistent with the increased abundances of *STAR* and *HSD3B1* mRNA in the porcine CL on Day 14 of pregnancy^39^.

The present results indicated that PROK1 increased capillary-like structure formation by luteal endothelial cells, which stimulated angiogenesis in the porcine CL. Interestingly, angiogenesis in the CL is more intensive than in any other organ and similar to that in a rapidly growing tumor^1^. Newly formed blood vessels are adjacent to the membranes of luteal cells (60%) or close to the interstitial space (29%) because luteal cells consume two to six times more oxygen per unit weight than the liver, the kidneys, or even the heart^1^. During the mid-luteal phase and early pregnancy, when PROK1 expression in the porcine CL is high, angiogenesis is an intensive process that ensures luteal tissue with nutrients and substrates for P4 synthesis. Our findings agree with previous reports indicating PROK1 as a mitogen for endothelial cells^18,22,23,40,41^. In endocrine glands, it has been shown that PROK1 regulates microvessel formation by supporting endothelial cell survival, migration, and fenestration^18^. Moreover, an in vivo study confirmed the angiogenic role of PROK1 because administering PROK1 into rat ovaries intensified angiogenesis^9^. Our findings also suggest that PROK1 regulates angiogenesis indirectly by increasing the luteal gene expression of the angiogenic factor *ANG* and the secretion of VEGFA by porcine luteal tissue. In the porcine CL, the abundance of VEGFA mRNA and protein is elevated during the mid-luteal phase of the estrous cycle and drops at the onset of luteolysis^8^. Similarly, in bovine CL, the levels of *VEGFA* mRNA tend to decrease during the late luteal phase and in the regressing CL^27^. However, the results of the present study showed that the protein abundance of PROK1 in the porcine CL was not changed in the analyzed days of the estrous cycle, whereas in the bovine CL, the mRNA levels of *PROK1* increase at the onset of luteolysis^27^. Intriguingly, our results showed that in cyclic pigs, PROK1 increased VEGFA secretion by porcine luteal tissue at the onset of luteolysis, but in pregnant pigs, PROK1 stimulated VEGFA secretion by luteal tissue at both the time corresponding to the luteal phase (Day 12) and the time corresponding to the onset of luteolysis (Day 14). This, in turn, may suggest different roles for PROK1 depending on the physiological state. PROK1 may contribute to processes controlling luteolytic events in the regressing CL, whereas during pregnancy, PROK1 may support mechanisms regulating angiogenic processes in the functioning CL. Moreover, our results are consistent with previous reports indicating that PROK1 increases *VEGFA* mRNA expression in bovine luteal steroidogenic cells^26^. In addition, a similar relationship between PROK1 and VEGFA secretion has been reported for endometrial explants in pigs^22^.

**Figure 8:**
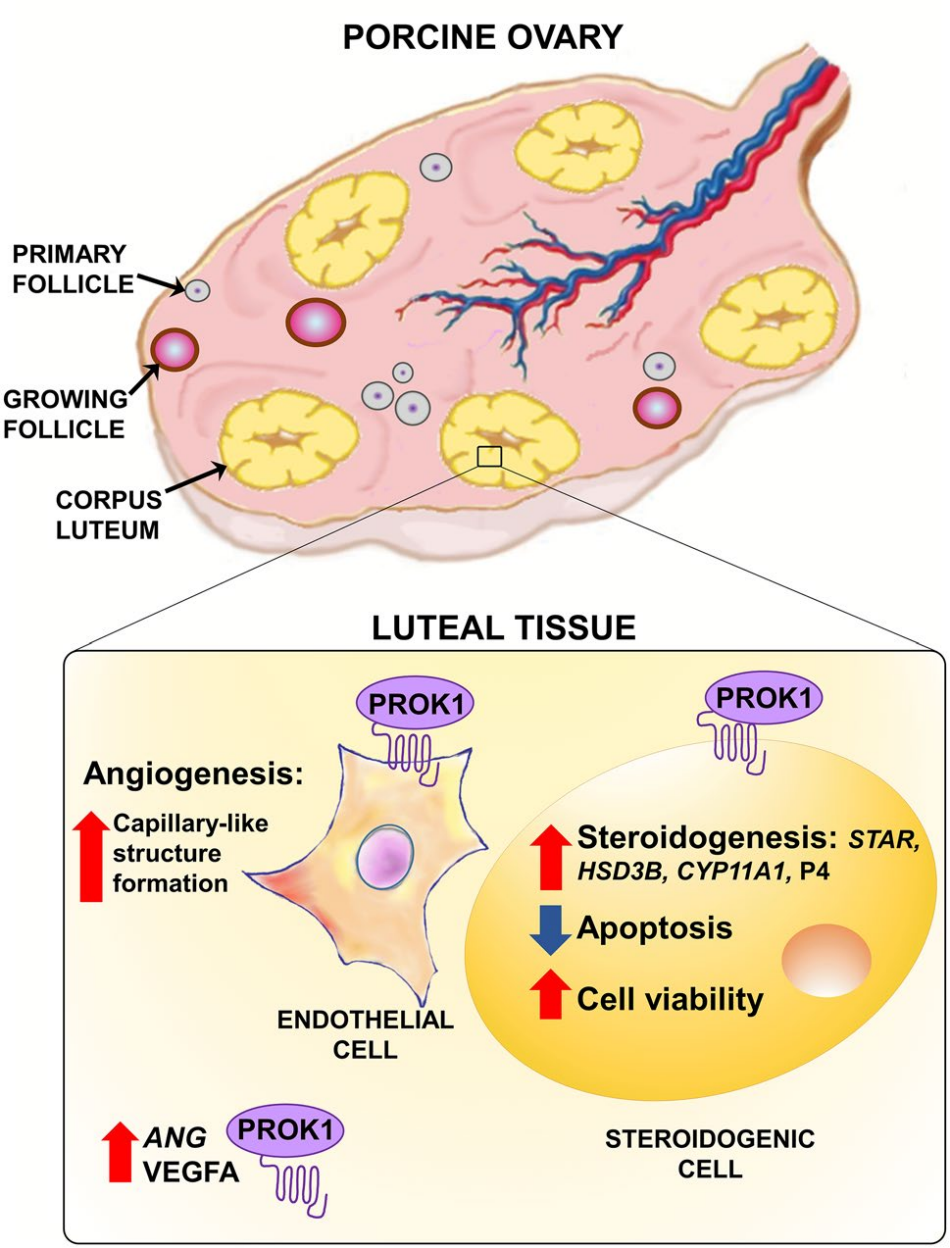
Proposed mechanism of prokineticin 1 (PROK1) for regulating the porcine corpus luteum function during the mid-luteal phase of the estrous cycle (Day 12 of the estrous cycle) and early pregnancy (the maternal recognition of pregnancy and the rescue of CL from regression, Days 12 and 14 of pregnancy). A greater abundance of PROK1, acting through the related receptor (PROKR1), increases the viability of luteal cells and reduces luteal cell apoptosis, as well as promotes steroidogenesis by increasing the expression of genes involved in progesterone (P4) synthesis: steroidogenic acute regulatory protein (*STAR*), hydroxy-delta-5-steroid dehydrogenase, 3 beta- and steroid delta-isomerase 1 (*HSD3B1*), and cytochrome P450 family 11 subfamily A member 1 (*CYP11A1*), and elevating P4 secretion. PROK1–PROKR1 signaling also promotes angiogenesis by stimulating the capillary-like structure formation by luteal endothelial cells and increasing vascular endothelial growth factor A (VEGFA) secretion and angiogenin (*ANG*) mRNA expression in luteal tissue. PROKR2 mRNA and protein are expressed in the porcine CL and also may have a putative role in effects of PROK1 on CL. A scheme of ovary was prepared based on a cartoon adapted and modified from^48^.

In conclusion, our study thoroughly elucidates the role of PROK1 in the porcine corpus luteum during the mid- and late luteal phase of the estrous cycle and early pregnancy. Our results indicate that PROK1, which is abundantly expressed in the porcine corpus luteum at the mid-luteal phase of the estrous cycle and early pregnancy, participates in processes related to CL function, such as progesterone synthesis, luteal cell apoptosis and viability, and angiogenesis (Fig. 8). Our results suggest that during the rescue of the corpus luteum from luteolysis on Days 12–14 of pregnancy, an increase in the abundance of the PROK1 protein, acting via PROKR1, increases the viability of luteal cells, reduces luteal cell apoptosis, promotes steroidogenesis by increasing the expression of genes involved in P4 synthesis (*STAR*, *HSD3B1*, and *CYP11A1),* and elevates P4 synthesis by luteal tissue. PROK1–PROKR1 signaling also stimulates capillary-like structure formation by luteal endothelial cells and promotes angiogenesis by increasing VEGFA secretion and angiogenin mRNA expression in luteal tissue. Therefore, we concluded that PROK1 is an important regulator of porcine CL function during the mid-luteal phase of the estrous cycle and early pregnancy.

## Materials and methods

The ovaries used in our research were collected from animals bound for commercial slaughter and meat production. The use of animals was in accordance with the Act of 15 January 2015 for the Protection of Animals Used for Scientific or Educational Purposes, Directive 2010/63/EU of the European Parliament and the Council of 22 September 2010 on the protection of animals used for scientific purposes and conducted in accordance with the national guidelines for agricultural animal care, and the ARRIVE guidelines. Experiments were not carried out on human tissue or cells samples.

### Experiment 1. The expression profiles of PROK1 and its receptors (PROKR1 and PROKR2) in the porcine corpus luteum during early pregnancy and the estrous cycle

Gilts of similar age and genetic background from one commercial herd were observed for the onset of estrus. After two natural estruses, the animals were randomly divided into two groups: pregnant and cyclic. Gilts assigned to the pregnant group were artificially inseminated at 12 and 24 h after the onset of estrus (Day 0). Animals were slaughtered in a local slaughterhouse on Day 9, 12, or 14 of pregnancy or the estrous cycle (n = 4–12 per group; no animals were pooled for replicates; each biological replicate represents a different animal). Reproductive status (cyclic/pregnant) was assessed based on the presence and morphology of conceptuses and the concentration of progesterone in plasma from jugular vein blood (Supplementary Fig. 1). The estrous cycle stage was confirmed by estrus observation and assessment of the ovarian morphology. Gilts that were fertilized but nonpregnant were excluded from the experiments. Luteal tissue samples were collected from CL separated from ovaries and snap-frozen in liquid nitrogen. The luteal expression of PROK1, PROKR1, and PROKR2 genes and proteins was determined by using Western blotting and quantitative PCR (qPCR; real-time RT‒PCR), as described in the “Gene expression analyses” and “Protein expression analyses” sections in the Supplementary Information.

### Experiment 2. Immunolocalization of PROK1, PROKR1, and PROKR2 proteins in the porcine corpus luteum

Prokineticin 1 and its receptors (PROKR1 and PROKR2) were immunolocalized in the porcine corpora lutea on Days 12 and 14 of the estrous cycle and pregnancy (n = 3 per group; each biological replicate represents a different animal) as described previously^22^ with some modifications. A full description of the procedure is provided in the Supplementary Information.

### Experiment 3. The effects of PROK1 on the synthesis of progesterone by porcine luteal explants in vitro

The effects of PROK1 on the expression of genes involved in steroidogenesis as well as on progesterone synthesis by luteal tissue explants were studied using an in vitro model of precision-cut luteal slices as described previously^42^, with some modifications. Briefly, the ovaries were collected from gilts on Days 12 and 14 of the estrous cycle or pregnancy (n = 4–7 per group; no animals were pooled for replicates; each biological replicate represents a different animal), placed in medium M199 (cat. no. 2525; Merck, Darmstadt, Germany) supplemented with antibiotics (penicillin, streptomycin; P0781, Sigma–Aldrich, Saint Louis, USA) and 0.1% (wt/vol) bovine serum albumin (BSA; ICN Biomedicals, Costa Mesa, USA) and transported to the laboratory on ice. Then, a corpus luteum was separated from the rest of the ovary and cut out using a tissue coring tool. Luteal tissue was transferred onto a Krumdick Tissue Slicer (K&F Research, Birmingham, AL, USA). Tissue explants of similar dimensions and weight (~ 180 μm thick, 8 mm diameter, 4–5 mg wet weight) were cut and collected in a Petri dish filled with medium M199. The luteal explants were then transferred to 6-well plates (one luteal slice and 4 mL of medium per well). For one hour, the explants were preincubated in medium with or without a PROKR1 antagonist (2-(5-(4-fluorobenzyl)-1-(4-methoxybenzyl)-1,4,5,6-tetrahydro-4,6-dioxo-1,3,5-triazin-2-ylamino)-ethyl)-guanidine; PC7, 10 μM). Then, the medium was changed, and the explants were incubated for 18 h in a humidified atmosphere containing 95% air and 5% CO_2_ at 37 °C with gentle shaking with either vehicle (control, 0.01% ethanol) or PROK1 (40 nM; cat. no. 100–44, PeproTech, Rocky Hill, NJ, USA) in the presence or absence of a PROKR1 antagonist (PC7, 10 μM). The IC50 for PROKR1 is 36 nM, whereas that for PROKR2 is 4400 nM^43^. The amino acid sequence similarity of PROKR1 between pigs and mice (the species for which the antagonist was tested for the first time) was 83.46%. The antagonist was kindly donated by Dr. Gianfranco Balboni from the University of Cagliari in Cagliari, Italy. The doses of PROK1 and PC7 were chosen based on previous studies^22,23,44^. Following incubation, the luteal explants and culture media were snap-frozen in liquid nitrogen and transferred to -80 °C for future analyses. The expression of genes involved in P4 synthesis (*CYP11A1*, *HSD3B1*, and *STAR*) in the luteal explants was investigated by qPCR, as described in the section “Gene expression analyses” provided in the Supplementary Information. The concentration of P4 secreted by precision-cut luteal slices was determined in collected culture medium by radioimmunoassay (RIA) as described in the Supplementary Information. The viability of luteal explants before and after incubation and in response to the different treatments was analyzed by Alamar Blue assay as described in the Supplementary Information.

### Experiment 4. The effect of PROK1 on the apoptosis and viability of luteal cells

#### Isolation and culture of porcine luteal cells

To investigate the effects of PROK1 on the apoptosis/viability of luteal cells, we isolated steroidogenic cells from porcine CL. Ovaries were collected from gilts on Day 12 of the estrous cycle (n = 11; no animals were pooled for replicates; each biological replicate represented a different animal). Luteal tissue was dissected from the surrounding ovarian tissue and mechanically fragmented using a scalpel. Subsequently, the tissue was enzymatically digested in 0.07% (w/v) collagenase (cat. no. C0130; Sigma–Aldrich) solution in M199 medium supplemented with 1% BSA for 70 min at 37 °C. The total cell suspension contained luteal and endothelial cells. The post-digested cell suspension was then filtered through gauze. Luteal cells were separated from the obtained supernatant through a series of centrifugations (45 × g, 29 × g, and 16 × g each for 10 min). The homogeneity of the isolated cells was confirmed by immunodetection of the luteal cell marker HSD3B1. Detailed information on luteal cell characteristics is provided in the Supplementary Information.

#### Apoptosis assay—Fluorescence-activated single cell sorting (FACS) against propidium iodide and FITC-annexin V by flow cytometry

Luteal cells were seeded into 6-well plates at a concentration of 4 × 10^5^ in M199 medium with 10% newborn calf serum (NCS; Sigma–Aldrich, Saint Louis, USA). After 24 h, the cells were treated with vehicle or 40 nM PROK1 in the presence or absence of 1 µM PC7 for 20 h. After incubation, the cells were detached enzymatically using Accutase solution (cat. no. A6964, Merck), and apoptosis was assessed using the flow cytometer FACS Aria II (BD Bioscience, Franklin Lakes, NJ, USA) and a FITC Annexin V/Dead Cell Apoptosis Kit (cat. no. V13242, Invitrogen, Paisley, UK) according to the manufacturers’ instructions. The cell populations were classified into three groups: live cells, apoptotic cells, and dead cells. The experiment was repeated 11 times (number of animals = 11).

### Experiment 5. The effect of PROK1 on luteal angiogenesis in vitro

To determine whether PROK1 participates in angiogenesis in the porcine corpus luteum, an in vitro model of a capillary-like structure formation assay and a model of precision-cut luteal slices were used.

#### Isolation and culture of porcine endothelial cells from the corpora lutea

Endothelial cells were isolated during the luteal cell isolation procedure (described in Experiment 4). For isolation of endothelial cells, the supernatant obtained during the first three centrifugations was collected and kept on ice. The collected supernatant was filtered (filter with a pore diameter of 15 µm) and centrifuged at 300 × g for 10 min. Subsequently, the cell precipitate was suspended in culture medium MCDB 131 (cat. no. M8537, Sigma–Aldrich, Saint Louis, USA), including 10% NCS and 50 μg/mL endothelial cell growth supplement (cat. no. E2759, Merck, Darmstadt, Germany), and cells were seeded onto a sterile plate. The cells were cultured in a humidified atmosphere containing 5% CO_2_ at 37 °C. The homogeneity of the isolated cells was confirmed by examining the cell cultures for the presence of the endothelial cell marker vWF. Detailed information on the endothelial cell characteristics is provided in the Supplementary Information.

#### Capillary-like structure formation assay

A capillary-like structure formation assay was performed as described previously^22,45^. Briefly, isolated endothelial cells from the corpora lutea (3 × 10^5^ cells/mL) were suspended in MCDB-131 medium with or without PC7 (1 μM) and preincubated for 20 min in a humidified atmosphere containing 5% CO_2_ at 37 °C. Subsequently, the cells were treated with PROK1 (40 nM) or vehicle (the control). The cells were then plated onto μ-Slide angiogenesis plates (cat. no. 81506; Ibidi GmbH, Gräfelfing, Germany) covered with growth factor-reduced Matrigel (cat. no. 354230; Corning, NY, USA) and incubated for 5 h at 37 °C in a humidified atmosphere containing 95% air and 5% CO_2_ in a Zeiss Axio Observer System (Carl Zeiss Microscopy). Single wells were photographed at 30-min intervals. The photographs were analyzed using ImageJ software with an angiogenesis plug-in^46^. This software is able to measure and quantify morphological structures formed by endothelial cells. The parameters describing the formation of the capillary-like networks were defined as described earlier^47^. Pixels with at least three neighbors were defined as nodes. Junctions were referred to as nodes or fused nodes. Elements limited by two junctions/nodes were defined as the segments. The branches consisted of elements delimited by a junction and one extremity, whereas the pieces of three, limited by two junctions (but not exclusively associated with one branch), were considered master segments. At least three linked master segments were defined as master junctions. Areas limited by the segments or master segments were considered meshes. The experiment was repeated 10 times (number of animals = 10) in duplicate (each treatment was duplicated).

#### *The effect of PROK1 on the expression of genes associated with angiogenesis and VEGFA secretion in porcine luteal explants *in vitro

We used precision-cut luteal slices described in Experiment 3 to evaluate whether PROK1 may be involved in angiogenic changes in the porcine CL (n = 4–7 per group; no animals were pooled for replicates; each biological replicate represented a different animal). The expression of genes associated with angiogenesis, including angiogenin (*ANG*), angiopoietin 2 (*ANGPT2*), fibroblast growth factor 2 (*FGF2*), fms-related receptor tyrosine kinase 1 (*FLT1*), and kinase insert domain receptor (*KDR*), and the secretion of VEGFA in response to PROK1 treatment were evaluated. The level of VEGFA in the culture medium was determined by enzyme-linked immunosorbent assay (ELISA; Supplementary Information), whereas the gene expression was determined by qPCR, as described in the section “Gene expression analyses” provided in Supplementary Information.

### Statistical analyses

The results obtained in Experiment 1 were assessed using two-way ANOVA, followed by Bonferroni multiple comparison post-test. The results from Experiment 3, studying the effect of PROK1 on the expression of angiogenic genes and VEGFA secretion (Experiment 5) and assessment of P4 concentration in jugular vein blood plasma collected from cyclic and pregnant gilts were evaluated by two-way ANOVA, followed by Tukey’s post-test. The results obtained from the apoptosis assay (Experiment 4), capillary-like structure formation assay (Experiment 5) and luteal explants viability after treatment were assessed using one-way ANOVA, followed by Tukey's multiple comparisons post-test. The results from luteal explants viability before and after incubation were analyzed using T-tests. Differences were considered statistically significant at a 95% confidence level (*p* < 0.05). All statistical analyses were conducted using GraphPad PRISM v. 9.0.0 software (GraphPad Software Inc., San Diego, CA, USA).

### Ethics approval

Ovaries used in our research were collected from animals bound for commercial slaughter and meat production. The use of animals was in accordance with the Act of 15th of January 2015 on the Protection of Animals Used for Scientific or Educational Purposes, Directive 2010/63/EU of the European Parliament and the Council of 22nd of September 2010 on the protection of animals used for scientific purposes and conducted in accordance with the national guidelines for agricultural animal care, and the ARRIVE guidelines. Experiments were not carried out on human tissue/cells samples.

## Supplementary Information

Supplementary Information.

## Data Availability

The datasets used and/or analyzed during the current study are available from the corresponding author on reasonable request.
