# Supplementary material for: Prokineticin 1 is a novel factor regulating porcine corpus luteum function
Source: Sci Rep. 2023 Mar 29;13:5085. doi: 10.1038/s41598-023-32132-3 (PMC10060428; doi:10.1038/s41598-023-32132-3)
Supplement: Supplementary file 1 — Supplementary Information. [file 41598_2023_32132_MOESM1_ESM.pdf]

Supplementary Dataset

Prokineticin 1 is a novel factor regulating the porcine corpus luteum function

Monika Baryla[1], Ewelina Goryszewska-Szczurek[1], Piotr Kaczynski[1], Gianfranco Balboni[2], Agnieszka Waclawik[1]*

[1] Institute of Animal Reproduction and Food Research, Polish Academy of Sciences, Tuwima 10, 10-748 Olsztyn, Poland.

[2]Department of Life and Environmental Sciences, University of Cagliari, Cittadella Universitaria, 09042 Monserrato (CA), Italy.

*Corresponding author:
Agnieszka Waclawik

Institute of Animal Reproduction and Food Research, Polish Academy of Sciences, Tuwima 10, 10-748 Olsztyn, a.waclawik@pan.olsztyn.pl

Major Category: Biological Sciences
Minor Category: Reproductive Biology

Supplementary Methods:

Immunohistochemistry procedure

Prokineticin 1 and its receptors (PROKR1 and PROKR2) were immunolocalized in the porcine corpora lutea on Days 12 and 14 of the estrous cycle and pregnancy as described previously[1] with some modifications. Briefly, corpora lutea dissected from the ovaries were fixed in 4% paraformaldehyde (PFA) and embedded in paraffin. Paraffin-embedded tissue was cut into 5 µm sections on a microtome. Tissue sections were placed onto glass slides and dewaxed with Neo Clear® xylene substitute (cat. no. 109843; Merck, Darmstadt, Germany) and rehydrated in decreasing ethanol gradient (100, 70, and 50%). Tissue antigens were retrieved by heating sections in 10 nM solution of citrate buffer (pH=6) for 15 minutes. Then, tissue sections were incubated in 3% hydrogen peroxidase in methanol for 30 minutes. Afterwards, sections were treated with SEA BLOCK Blocking Buffer (cat. no. 37527; Thermo Fisher Scientific, Waltham, MA, USA) to block unspecific reactions with antibodies. Sections were incubated overnight at 4° C with primary anti-PROK1, anti-PROKR1 and anti PROKR2 antibodies (Supplementary Table 1). Afterwards, secondary horseradish peroxidase-conjugated goat anti-rabbit IgG antibodies (1:200; Supplementary Table 1) were applied to the sections for 45 minutes at room temperature. Sections were then incubated for 30 minutes with a mixture of reagents A and B (cat. no. PK-6102; Vector Laboratories Inc., Burlingame, CA, USA) in TRIS-buffered saline to activate conjugated peroxidase. Immune complexes were visualized using 3,3'-diaminobenzidine tetra-hydrochloride hydrate (cat. no. D5637, Sigma–Aldrich, Saint Luis, USA). Subsequently, sections were slightly counterstained with hematoxylin and dehydrated with a rising gradient of alcohols (70, 96, and 100%). Sections were incubated with xylene substrate (twice for 10 and 15 minutes) and mounted using DPX (cat. no. 44581, Fluka, Buchs, Switzerland). Negative controls were performed with normal rabbit IgG (I-1000-5; Vector Laboratories) diluted at the same protein concentration as the primary antibodies, omitting the primary antibodies (PROK1 and PROKR2), and by using blocking peptide (PROKR1, cat. no. LS-E29168, LSBio, Seattle, WA, USA). Tissue sections were observed under a light microscope (Olympus Provis AX70, Tokyo, Japan) and photographed.

Characteristics of luteal cells

Luteal cells homogeneity was confirmed by immunodetection of HSD3B1 in the isolated cells. The cells were seeded on Cell Imaging Coverglasses (cat. no. 003074206; Eppendorf, Hamburg, Germany) at a density of 5 × 104. The attached cells were washed with PBS before fixing in 2% PFA for 15 min. The cells were then washed with PBS and blocked with newborn donkey serum (cat. no. 017-000-121, Jackson ImmunoResearch, Cambridgeshire, UK) for 1 hour at room temperature. Subsequently, the buffer was removed, and cells were incubated overnight with primary anti-HSD3B1 antibodies at 4°C (Supplementary Table 1). Afterwards, the cells were washed with PBS and incubated with secondary antibodies for 1 hour at room temperature (Supplementary Table 1). Negative controls were performed by omitting the primary antibodies. Cell nuclei were counterstained with DAPI (cat. no. ab104135; Abcam, Cambridge, UK). Slides were mounted with anti-fade fluorescence mounting medium (cat. no. ab104135, Abcam, Cambridge, UK). Fluorescent images of stained cells were captured using an Olympus microscope (Olympus BX-40) equipped with filters set for CY3.

Characteristics of endothelial cells

The homogeneity of isolated cells was confirmed by examining cell culture for the presence of the endothelial cell marker, von Willebrand factor (vWF). Isolated cells after the first passage were cultured for 3 days on Cell Imaging Coverglasses at a seeding density of 1.5×10^5 cells/mL. The culture medium was removed, and cells were washed with PBS. Then, cells were fixed in 2% PFA for 15 minutes at room temperature. Subsequently, cells were washed and blocked with SEA BLOCK Blocking Buffer for 1 hour at room temperature. The cells were washed and then incubated overnight with primary anti-vWF antibodies at 4°C (Supplementary Table 1). The next day, cells were incubated with secondary anti-rabbit antibodies (Supplementary Table 1) for 1 hour at room temperature. Negative controls were performed by replacing the primary antibodies with normal rabbit IgG diluted to the antibody concentrations (Supplementary Table 1). Actin filaments were stained with CytoPainter Phalloidin-iFluor 488 Reagent (cat. no. ab176753, Abcam, Cambridge, UK). Additionally, to visualize cell nuclei, counterstaining with DAPI was performed. Slides were mounted with anti-fade fluorescence mounting medium. Fluorescent images of stained cells were captured using an Olympus microscope (Olympus BX-40) equipped with filters set for CY3 and iFluor488.

Gene expression analyses

The expression of *PROK1*, *PROKR1*, and *PROKR2* genes in porcine CL on Days 9, 12, and 14 of pregnancy and the estrous cycle, as well as the expression of *CYP11A1, HSD3B1, STAR, ANG, ANGPT2, FLT1*, and *KDR* genes in porcine luteal explants in response to PROK1 treatment, was determined by qPCR, as described previously[1]. Briefly, total RNA was isolated from collected samples using commercially available kits (ex vivo samples—A&A Biotechnology, Gdansk, Poland; in vitro samples—Qiagen, Hilden, Germany) according to the manufacturer's protocols. The quality and quantity of isolated RNA were assessed using Agilent Bioanalyzer (Agilent Technologies, Santa Clara, USA) and NanoDrop1000 (Thermo Fisher Scientific, Waltham, USA). Total RNA was then reverse-transcribed using a high-capacity cDNA reverse transcription kit (Life Technologies, Carlsbad, USA). Synthesized cDNA was used as a template in real-time PCR. The expression of the studied genes was analyzed using TaqMan Master Mix with TaqMan probes (Life Technologies, Supplementary Table 2) according to the manufacturer's protocol. The PCR programs for all genes were as follows: initial denaturation (95°C, 10 min) followed by 40 cycles of denaturation (95°C, 15 sec), annealing, and elongation (60°C, 1 min). All real-time PCR reactions were performed with Applied Biosystems 7900HT Real-Time PCR system (Life Technologies). Gene expression was estimated using real-time PCR Miner software[2]. The following formula was used to calculate gene expression from values generated by PCR Miner software: 1/(1+Average Efficiency)^CT. The stability of reference genes actin beta (*ACTB*), cyclophilin A (*PPIA*), and glyceraldehyde 3-phosphate dehydrogenase (*GAPDH*) was assessed separately by NormFinder[3] for ex vivo and in vitro experiments. The results of gene expression in luteal tissue from Days 9, 12, and 14 of pregnancy and the estrous cycle were normalized against the geometric mean of *ACTB* and *PPIA*, whereas the gene expression in luteal explants collected after in vitro experiments was normalized against the geometric mean of *GAPDH* and *PPIA*. The geometric mean was calculated for each sample separately using the expression values of selected reference genes.

Protein expression analyses

Protein expression of PROK1, PROKR1, and PROKR2 in porcine corpora lutea on Days 9, 12, and 14 of pregnancy and the estrous cycle was evaluated by Western blotting analyses, as described previously[1] with some modifications. The luteal tissue samples were homogenized in a homogenization buffer (50 mM/L TRIS-HCl, pH 8.0; 150 mM/L NaCl; 1 mM/L EDTA) containing a protease inhibitor cocktail (Sigma–Aldrich). Total protein extracts (70 µg for PROK1; 50 µg for PROKR1 and PROKR2) were dissolved in SDS-gel loading buffer (50 mM Tris-HCl pH 6.8, 4% SDS, 20% glycerol, and 2% β-mercaptoethanol), heated at 95°C for 4 min. Protein samples were separated on Tris-Tricine SDS-PAGE (PROK1)[1] or 10% Tris-Glycine SDS-PAGE gels (PROKR1 and PROKR2). Separated proteins were electroblotted onto a 0.2 µm (PROK1) or 0.4 µm (PROKR1, PROKR2) polyvinylidene difluoride membrane (PVDF). Then, the membranes were blocked in 5% nonfat dry milk in Tris-buffered saline (TBS) containing 0.1% Tween 20 (TBS-T). Subsequently, the PVDF containing samples to determine the expression of PROK1 were divided into two parts (the upper part was subjected to detection of GAPDH expression, while the lower part was subjected to detection of PROK1 expression). Afterwards, the membranes were incubated overnight at 4°C with appropriate primary antibodies (Supplementary Table 1). Next, the incubated membranes were washed three times with fresh TBS-T and incubated with secondary anti-rabbit alkaline phosphatase-conjugated antibodies (Supplementary Table 1) for 90 min at room temperature. To determine GAPDH expression (PROKR1 and PROKR2), membranes were washed three times with fresh TBS-T, re-blocked in 5% nonfat dry milk in TBS-T, and then incubated again with primary antibodies (the blots were not stripped). Negative controls were obtained by replacing the primary antibodies with normal rabbit IgG (IgG; sc-2027, Santa Cruz Biotechnology, Inc., Heidelberg, Germany) used at the same concentration as the primary antibodies. For PROKR1 additional negative control was performed with the primary antibody pre-absorbed with a blocking peptide (LS-E29168, LS Bio). Immune complexes were visualized using a standard alkaline phosphatase visualization procedure and documented in ChemiDoc™ MP Imaging System (Bio-Rad, Hercules, CA, USA). Normalization of the results was performed by dividing the expression of the tested protein by the expression of GAPDH (for each sample separately).

Alamar Blue Viability assay

The effect of the time of incubation and treatment with PROK1 (40 nM), PC7 (10 µM) and PROK1+PC7 on the viability of the luteal explants was assessed by the Alamar Blue assay (cat. no. DAL 1025, Invitrogen, Waltham, MA, USA). Metabolically active living cells change the color of Alamar Blue from blue to pink, while dead or inactive cells do not change the original blue color. The measurement was performed according to the manufacturer's protocol. The ovaries were collected from gilts on Day 12 of the estrous cycle (n = 4). The luteal explants were prepared as described in the Experiment 3. To determine the initial viability (control T0), CL slice were pre-incubated for 1 h, then the medium was changed and incubated for 4 h with Alamar Blue. Whereas, to determine the viability after treatment, the CL explants were incubated with control (vehicle, control T18) or PROK1 (40 nM) for 14 h in the presence/absence of prokineticin receptor 1 (PROKR1) antagonist (PC7; 10 µM). After 14 h, Alamar Blue was added to the culture medium and incubated for a further 4 h (total incubation time was 18 h). After 4 h incubation with Alamr Blue, 300 µL of reaction mixtures were transferred to 96-well plates (100 µL per well). Absorbance measurement was performed using Epoch Microplate Spectrophotometer (Agilent BioTek, Santa Clara, CA, USA) at 570 and 600 nm. Medium (M199) with 10% Alamar Blue, without any CL explant, was used as blank for the absorbance measurements. The percentage of viability was calculated using the free software alamarBlue Colorimetric Calculator (https://www.bio-rad-antibodies.com/colorimetric-calculator-fluorometric-alamarblue.html).

Radioimmunoassay (RIA) for P4

The concentration of P4 secreted by precision-cut luteal slices to culture media and P4 concentration in plasma from blood collected post-mortem from the jugular vein were assessed using a commercially available RIA kit (cat. no. KIP1458, DIAsource Immuno Assays SA, Louvain-la-Neuve, Belgium) according to the manufacturer's protocol. The assay sensitivity was 0.19 pg/mL, and the intra-assay coefficient of variation (CV%) was 4.6% for calculations of P4 concentration in culture media and 4.03% for calculations in blood plasma.

Enzyme-linked Immunosorbent Assay (ELISA) of VEGFA

The level of VEGFA secreted to culture media by precision-cut luteal slices was determined by a commercially available ELISA assay (cat. no. ES25RB; Thermo Fisher Scientific, Waltham, USA). The measurement was performed according to the manufacturer's protocol with some modifications. The standard curve was extended by adding standards 10 and 5 pg/mL, and the calculated sensitivity was 5 pg/mL.

Supplementary Tables:

Supplementary Table 1. List of antibodies used in Western blot (WB), immunohistochemistry (IHC) and immunofluorescence experiments.

**Protein target**	**Name of antibody**	**Manufacturer, catalog no., or name of source**	**Species raised in monoclonal or polyclonal**	**Dilution Used**
PROK1	Anti-PROK1 antibody	Phoenix Pharmaceuticals, G-023-59 (Western blot)/ H-023-59 (Immunohistochemistry)	Rabbit, polyclonal	1:100 (WB)/ 1:150 (IHC)
PROKR1	Anti-PROKR-1 antibodies	LSBio LifeSpan Bioscience, LS-A6684	Rabbit, polyclonal	1:200 (WB)/ 1:150 (IHC)
PROKR2	Prokineticin receptor 2 (extracellular) antibody	Invitrogen, PA5-77688	Rabbit, polyclonal	1:200 (WB)/ 1:50 (IHC)
GAPDH	Anti-GAPDH antibody	Abcam, ab9485	Rabbit, polyclonal	1:2000
anti-rabbit AP	Anti-Rabbit IgG (whole molecule)–Alkaline Phosphatase antibody	Sigma-Aldrich, A3687	Goat, polyclonal	1:20000
Anti-rabbit Biotinylated	Anti-Rabbit IgG Antibody (H+L), Biotinylated	Vector Laboratories Inc, AB-1000	Goat, polyclonal	1:200
VWF	anti- von Wilebrand factor	Dako, A-0082	Rabbit, polyclonal	1:100
HSD3B1	Anti-HSD3B1	Abcam, ab55268	Mouse, monoclonal	1:100
Anti-mouse CY3	CY™ 3 AffiniPure Donkey Anti-Mouse IgG (H+L)	Jackson ImmunoResearch, 715-165-150	Donkey, polyclonal	1:2000
Anti-rabbit CY3	CY3-conjugated donkey anti-rabbit IgG	Jackson ImmunoResearch, 711165152	Donkey, polyclonal	1:2000

Supplementary Table 2. Assays used in real-time RT-PCR analyses.

Gene	Primer sequence/TaqMan Assay ID	GenBank accession no.
ACTB	**Ss03376081_u1**	AK237086.1
GAPDH	**Ss03373286_u1**	NM_001206359.1
PPIA	**Ss03394782_g1**	NM_214353.1
PROK1	**Ss04246562_m1**	NM_001172586.1
PROKR1	**ARGZGD3**	NC_010445.4
PROKR2	**ARWCXJZ**	NC_010459.5
HSD3B1	**Ss03391752_m1**	NM_001004049.2
CYP11A1	**Ss03384849_u1**	NM_214427.1
STAR	**Ss03381250_u1**	NM_213755.2
FGF2	**Ss03375809_u1**	AJ577089.1
FLT1	**Ss03375679_u1**	AJ245445.1
KDR	**Ss03376639_u1**	XM_013997943.2
ANG	**Ss03380513_u1**	NM_001044573.2
ANGPT2	**Ss03392365_m1**	NM_213808.1

Supplementary Figure 1

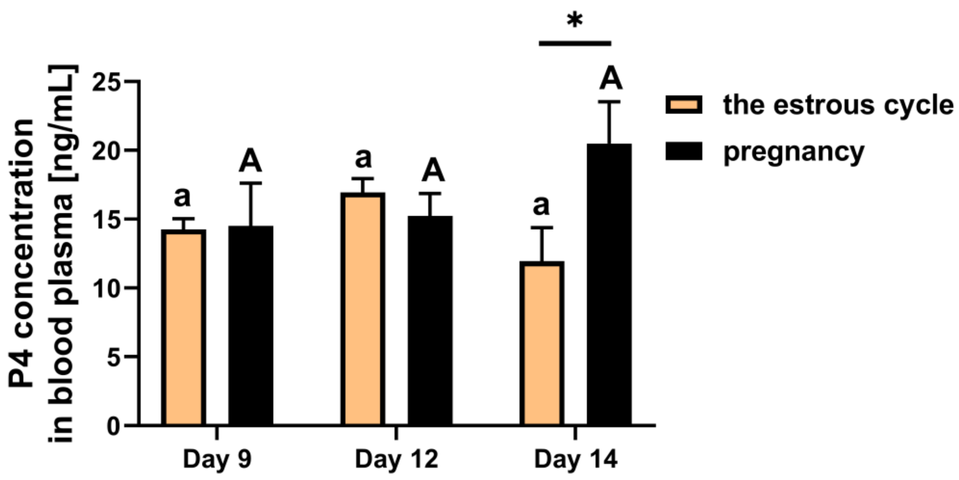

Supplementary Figure 1. Progesterone (P4) concentration in plasma from jugular vein blood collected post mortem from gilts on either Day 9, 12 or 14 of the estrous cycle or pregnancy. Data are presented as mean ± SEM. Lowercase letters (a) indicate no statistical significant differences between days of the estrous cycle, and capital letters (A) indicate no statistical significant differences between days of pregnancy. Statistical significance ($p<0.05$) of reproductive status has been marked with an asterisk (*).

Supplementary Figure 2

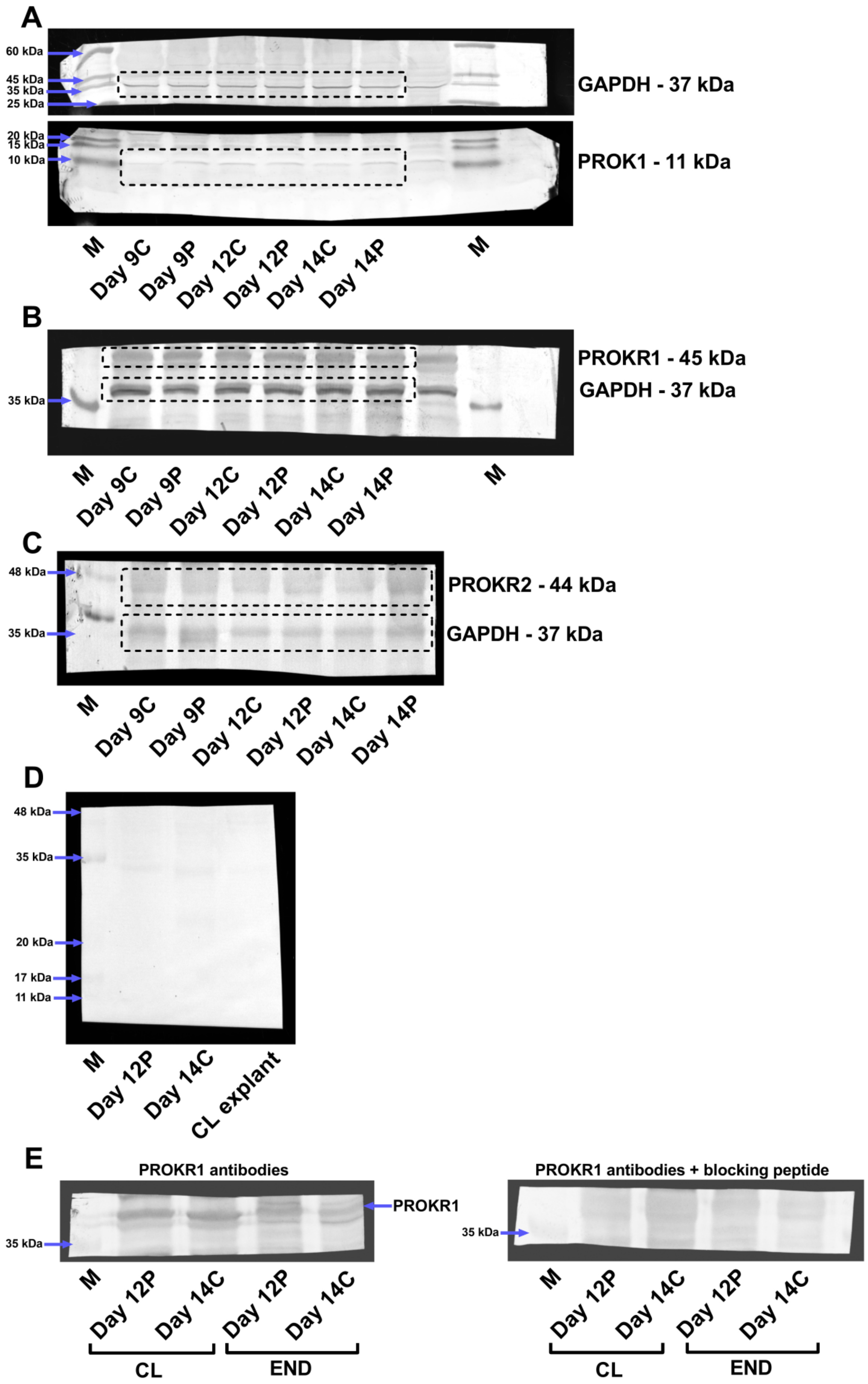

Supplementary Figure 2. Uncropped images of full length Western blot membranes presented in the Figure 1 for expression of prokineticin 1 (PROK1) (A); prokineticin receptor 1 (PROKR1) (B) and prokineticin receptor 2 (PROKR2) (C). Negative controls were obtained by: a replacement of the primary antibodies with normal rabbit immunoglobulin G used at the concentration corresponding to the protein concentrations of antibodies (D) and using the primary antibody for PROKR1 pre-absorbed with a blocking peptide (E). The membranes were cut prior to hybridization with the antibodies to reduce the consumption of the antibodies. Colorimetric method of Western blot detection in which membranes are photographed on the black background was used. M - Protein Molecular Weight Marker; CL – corpus luteum tissue; END – endometrium tissue.

Supplementary Figure 3

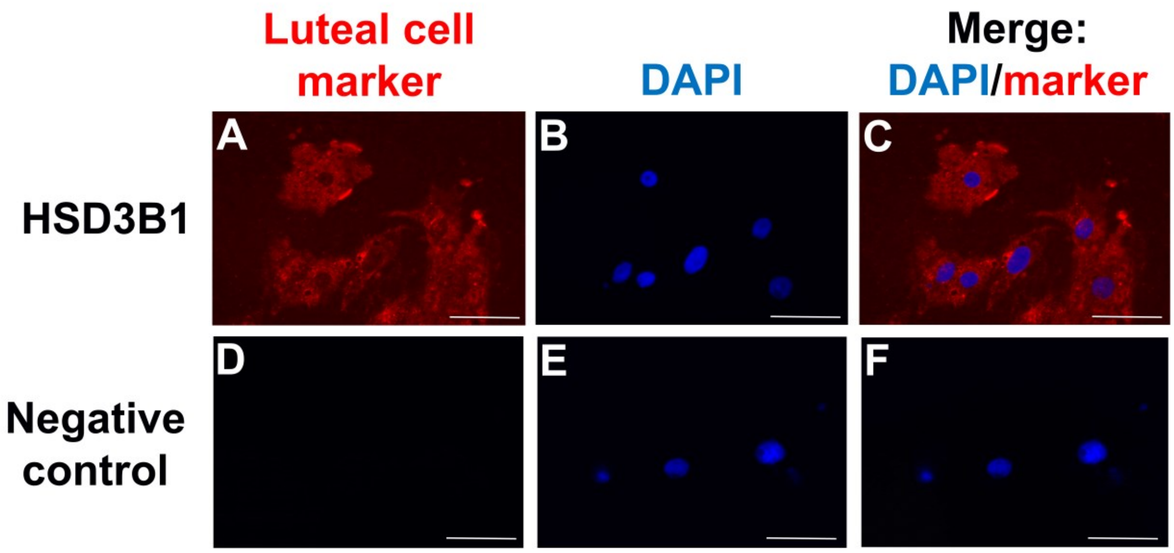

Supplementary Figure 3. Immunolocalization of (A) hydroxy-delta-5-steroid dehydrogenase, 3 beta- and steroid delta-isomerase 1 (HSD3B1) factor in the porcine luteal cells. Cell nuclei were stained with (B and E; blue) DAPI dye. Specificity of immunoreaction was determined by using (D) negative control – omitting the primary antibody. Photos merge (DAPI/marker) are shown in the last column (C, and F). Scale bars: 50 µm.

Supplementary Figure 4

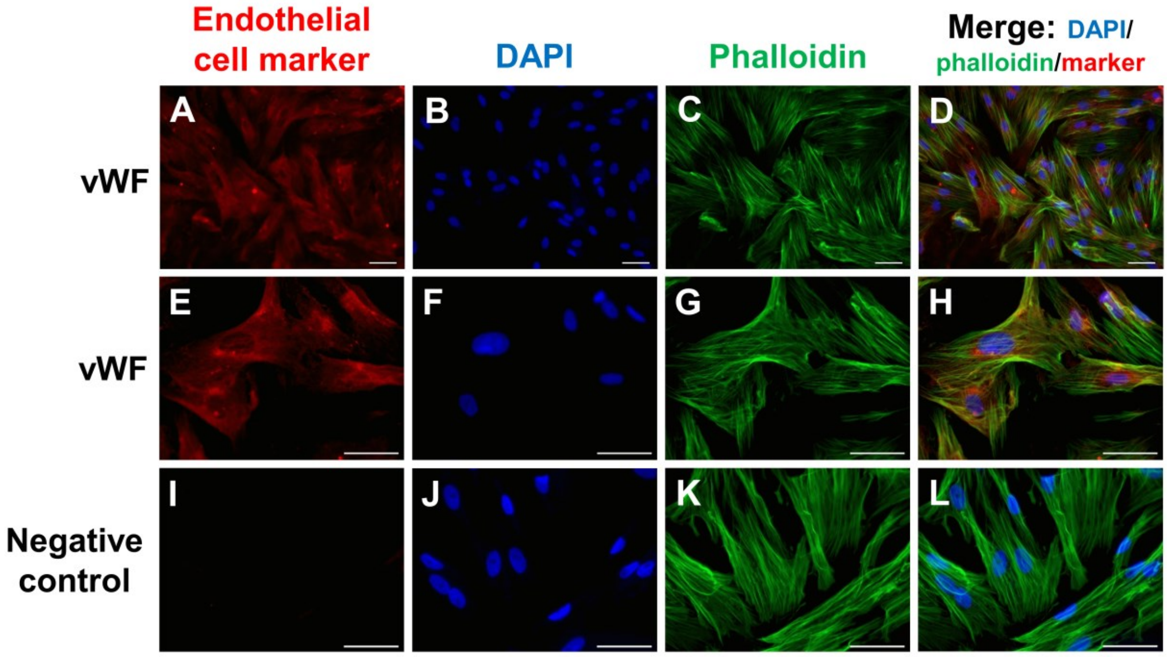

Supplementary Figure 4. Immunolocalization of von Willebrand (vWF) factor (A and E) in the porcine luteal endothelial cells. Cell nuclei were stained with DAPI dye (blue; B, F, and J) while the cytoplasm were stained with phalloidin (green; C, G, and K). Specificity of immunoreaction was determined by using (I) negative controls – normal rabbit IgG. Photos merge (DAPI/phalloidin/marker) are shown in the last column (D, H, and L). Scale bars: 50 µm.

Supplementary Figure 5

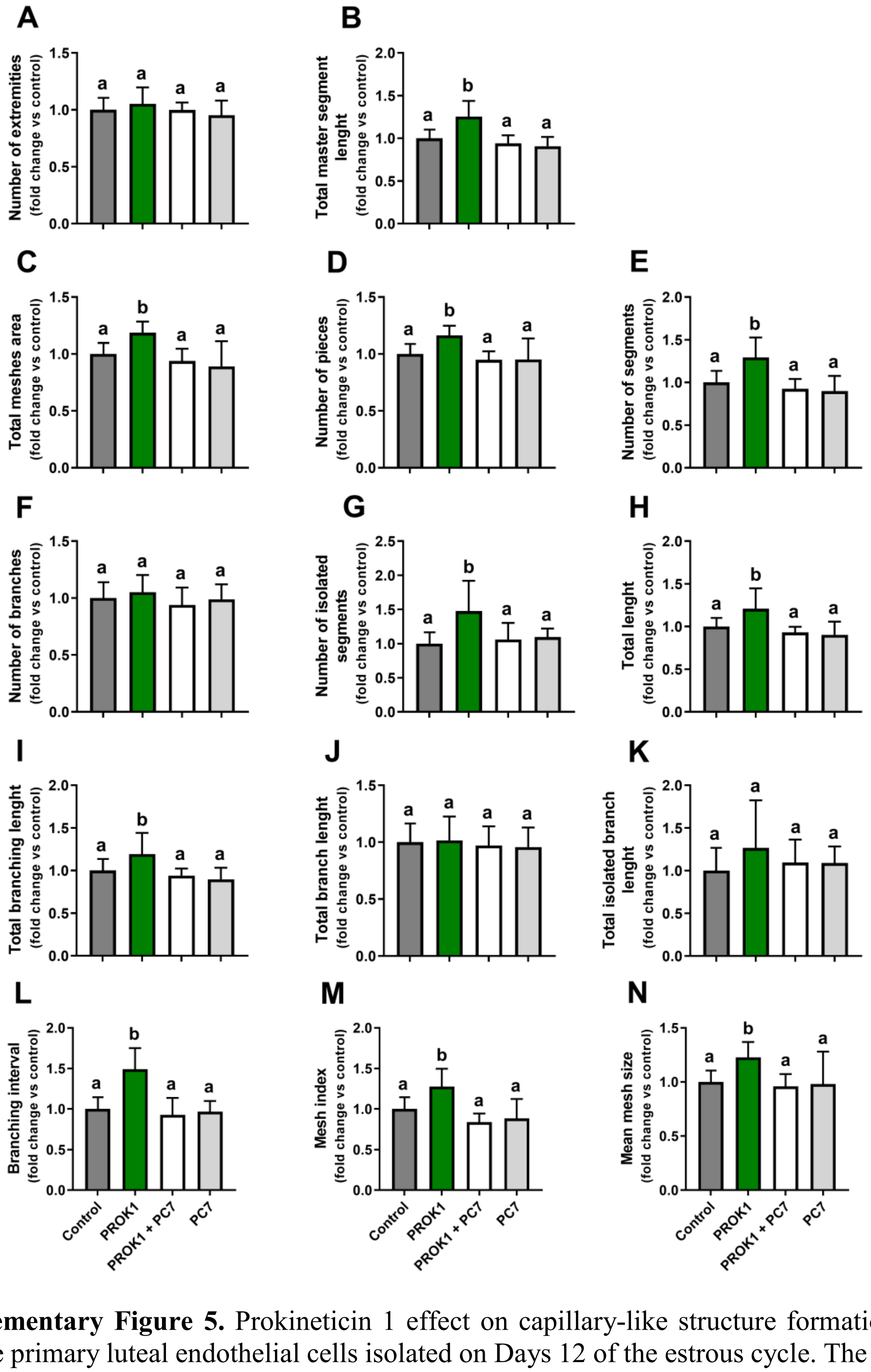

Supplementary Figure 5. Prokineticin 1 effect on capillary-like structure formation by the porcine primary luteal endothelial cells isolated on Days 12 of the estrous cycle. The effects of PROK1 on the parameters describing angiogenesis (excluding the results presented in Fig. 6), such as (A) the number of extremities, (B) total master segment length, (C) total meshes area, (D) number of pieces, (E) number of segments, (F) number of branches, (G) number of isolated segments, (H) total length, (I) total branching length, (J) total branch length, (K) total isolated branch length, (L) branching interval; (M) mesh index, and (N) mean mesh size were evaluated in the presence or absence of a PROK receptor 1 antagonist (PC7). Data are presented as the mean ± SEM of the fold change versus control. Different letters (a - b) indicate statistically significant differences ($p<0.05$).

Supplementary Figure 6

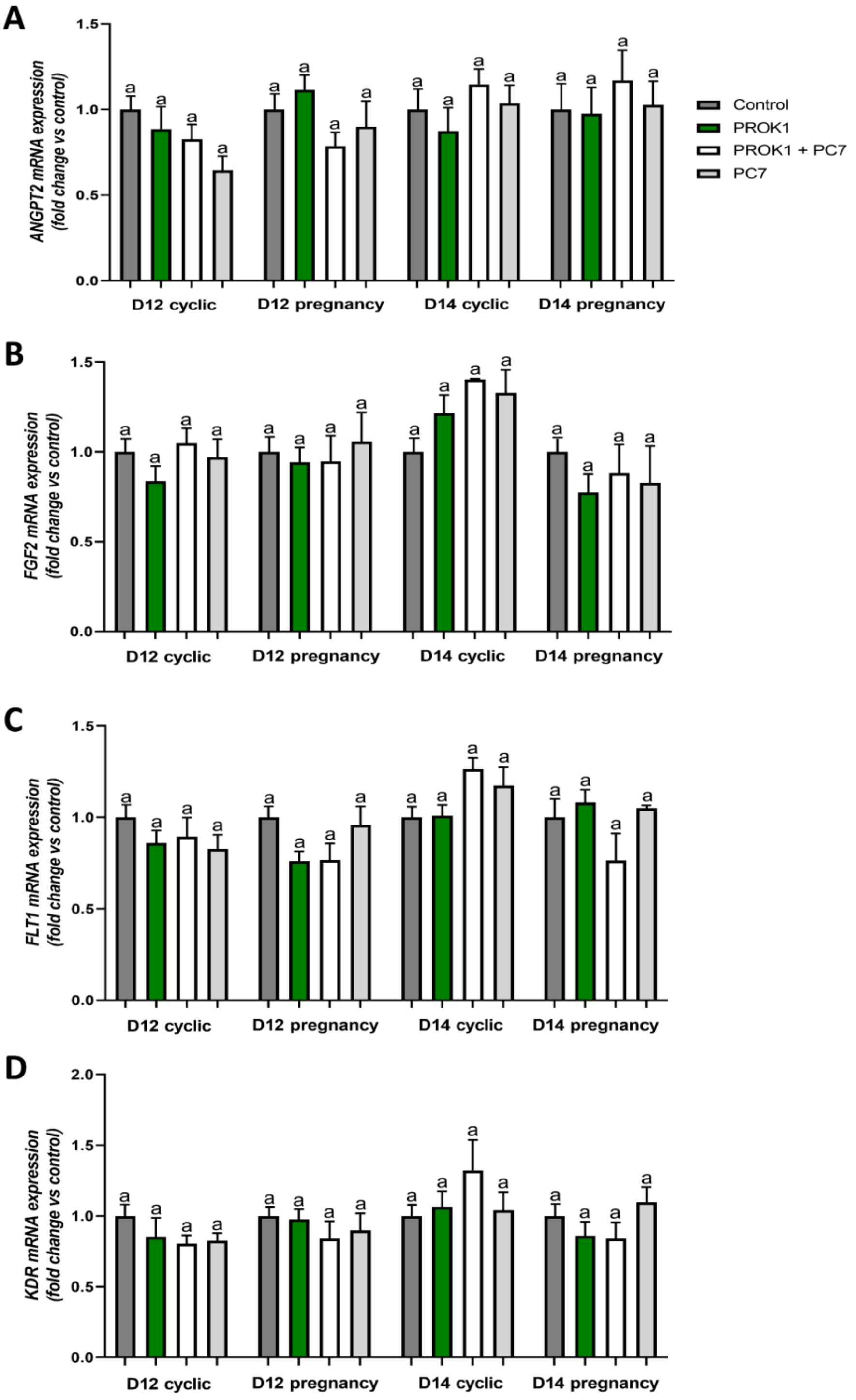

Supplementary Figure 6. The effect of prokineticin 1 (PROK1) on expression of genes associated with angiogenesis in the porcine luteal explants in vitro on Day 12 and 14 of estrous cycle or pregnancy: (A) angiopoietin 2 (*ANGPT2*), (B) fibroblast growth factor 2 (*FGF2*), (C) fms related receptor tyrosine kinase 1 (*FLT1*), and (D) kinase insert domain receptor (*KDR*). Precision-cut luteal slices were incubated with control (vehicle) or PROK1 (40 nM) for 18 h in the presence/absence of prokineticin receptor 1 (PROKR1) antagonist (PC7; 10 μM). Data are presented as means ± SEM. The same lowercase letters (a) indicate no statistically significant differences between treatments within groups on Days 12 (D12) and 14 (D14) of the estrous cycle or pregnancy.

References

1 Goryszewska, E., Kaczynski, P., Balboni, G. & Waclawik, A. Prokineticin 1-prokineticin receptor 1 signaling promotes angiogenesis in the porcine endometrium during pregnancy. *Biol Reprod* **103**, 654-668, doi:10.1093/biolre/ioaa066 (2020).

2 Zhao, S. & Fernald, R. D. Comprehensive algorithm for quantitative real-time polymerase chain reaction. *J Comput Biol* **12**, 1047-1064, doi:10.1089/cmb.2005.12.1047 (2005).

3 Andersen, C. L., Jensen, J. L. & Orntoft, T. F. Normalization of real-time quantitative reverse transcription-PCR data: a model-based variance estimation approach to identify genes suited for normalization, applied to bladder and colon cancer data sets. *Cancer Res* **64**, 5245-5250, doi:10.1158/0008-5472.CAN-04-0496 (2004).
